# The *Chlamydia trachomatis* Type III Secretion Chaperone Slc1 Engages Multiple Early Effectors, Including TepP, a Tyrosine-phosphorylated Protein Required for the Recruitment of CrkI-II to Nascent Inclusions and Innate Immune Signaling

**DOI:** 10.1371/journal.ppat.1003954

**Published:** 2014-02-20

**Authors:** Yi-Shan Chen, Robert J. Bastidas, Hector A. Saka, Victoria K. Carpenter, Kristian L. Richards, Gregory V. Plano, Raphael H. Valdivia

**Affiliations:** 1 Department of Molecular Genetics and Microbiology and Center for Microbial Pathogenesis, Duke University Medical Center, Durham, North Carolina, United States of America; 2 Department of Biochemistry, Duke University Medical Center, Durham, North Carolina, United States of America; 3 Department of Microbiology and Immunology, University of Miami Miller School of Medicine, Miami, Florida, United States of America; University of California San Francisco, United States of America

## Abstract

*Chlamydia trachomatis*, the causative agent of trachoma and sexually transmitted infections, employs a type III secretion (T3S) system to deliver effector proteins into host epithelial cells to establish a replicative vacuole. Aside from the phosphoprotein TARP, a *Chlamydia* effector that promotes actin re-arrangements, very few factors mediating bacterial entry and early inclusion establishment have been characterized. Like many T3S effectors, TARP requires a chaperone (Slc1) for efficient translocation into host cells. In this study, we defined proteins that associate with Slc1 in invasive *C. trachomatis* elementary bodies (EB) by immunoprecipitation coupled with mass spectrometry. We identified Ct875, a new Slc1 client protein and T3S effector, which we renamed TepP (Translocated early phosphoprotein). We provide evidence that T3S effectors form large molecular weight complexes with Scl1 *in vitro* and that Slc1 enhances their T3S-dependent secretion in a heterologous *Yersinia* T3S system. We demonstrate that TepP is translocated early during bacterial entry into epithelial cells and is phosphorylated at tyrosine residues by host kinases. However, TepP phosphorylation occurs later than TARP, which together with the finding that Slc1 preferentially engages TARP in EBs leads us to postulate that these effectors are translocated into the host cell at different stages during *C. trachomatis* invasion. TepP co-immunoprecipitated with the scaffolding proteins CrkI-II during infection and Crk was recruited to EBs at entry sites where it remained associated with nascent inclusions. Importantly, *C. trachomatis* mutants lacking TepP failed to recruit CrkI-II to inclusions, providing genetic confirmation of a direct role for this effector in the recruitment of a host factor. Finally, endocervical epithelial cells infected with a *tepP* mutant showed altered expression of a subset of genes associated with innate immune responses. We propose a model wherein TepP acts downstream of TARP to recruit scaffolding proteins at entry sites to initiate and amplify signaling cascades important for the regulation of innate immune responses to *Chlamydia*.

## Author Summary

*Chlamydia trachomatis* is an obligate intracellular bacterial pathogen that causes a range of human diseases of significant public health importance. To create a suitable replicative niche within its host, *Chlamydia* delivers effector proteins across mammalian membranes via a syringe-like apparatus termed a Type III secretion (T3S) system. The lack of a robust system for the molecular genetic manipulation of these pathogens has hindered progress in identifying and characterizing T3S effectors. In this study, we took a mass spectrometry-based approach to identify *Chlamydia* effector proteins based on their interaction with Slc1, an abundant T3S chaperone. We identified a previously uncharacterized protein, Ct875/TepP, as a new T3S effector and determined that TepP is phosphorylated upon translocation into host cells, leading to the recruitment of the host scaffolding protein Crk and presumably manipulating Crk-dependent signaling functions. Finally, we provide genetic confirmation of the role of TepP in recruiting Crk and in modulating the expression of genes involved in innate immune responses to *Chlamydia*. This study is the first example of genetic validation of the function of a T3S effector in *Chlamydia* and a new example of a bacterial effector that directly co-opts the oncoprotein Crk to modulate host cell signaling events.

## Introduction

The gram-negative bacterium *Chlamydia trachomatis* is the causative agent of trachoma, the leading cause of infectious blindness worldwide, and the major cause of bacterial sexually transmitted infections (STI) in the developed world [1]. Approximately 2.9 million cases of visual impairment and ∼1.17 million cases of blindness are attributed to ocular infections by *C. trachomatis* [2]. In the US, an estimated 2.8 million cases of *Chlamydia* STIs occur annually, imposing a significant burden on the public health system [3].

*Chlamydiae* have a biphasic life cycle that alternates between two unique developmental forms, the environmentally stable, infectious elementary body (EB), and the replicative, but non-infectious, reticulate body (RB) [4]. Infection starts with the attachment of EBs to host cell membranes. After inducing its own internalization, *C. trachomatis* rapidly modifies its endocytic vacuole to avoid fusion with lysosomes [5] and migrates to a perinuclear region of the cell [6], [7] where it undergoes a developmental transition to the RB form. Bacterial replication occurs within a membrane-bound vacuole called an inclusion, and mid-to-late in the infectious cycle, bacterial cell replication becomes asynchronous with RBs transitioning back to the EB form (for reviews, see [4], [8]). In late stages of infection, the inclusion occupies the bulk of the host cell cytoplasmic space and EBs are released to infect adjacent cells by cell lysis or extrusion of the inclusion [9].

Like many gram-negative bacterial pathogens, *Chlamydia* uses a type III secretion (T3S) system to deliver modulators (effector proteins) into their target host cell (reviewed in [10]). These effectors interfere with diverse host cellular processes including signaling, cytoskeletal rearrangements, and vesicle trafficking to enhance bacterial entry, establish a replicative niche and evade innate immunity [11]. The temporal manner in which effectors are secreted is likely to be important for the proper manipulation of host cell functions. For instance, the Translocated Actin Recruiting Protein (TARP) is delivered into the host cytoplasm within 5 min of bacterial attachment [12]. TARP facilitates invasion by mediating actin re-arrangements through the direct nucleation of F-actin polymerization and the recruitment of Rac-specific guanine nucleotide exchange factors [13], [14]. Another T3S effector, Ct694, is delivered into host cells during *Chlamydia* entry where it engages the cytoskeletal organizing protein AHNAK [15], although the timing of Ct694 translocation in relation to TARP is unknown. After entry, a new set of T3S effectors are synthesized and translocated to the inclusion membrane (Inc proteins) [16] where they mediate the recruitment of SNAREs, 14-3-3β, Rab proteins, signaling molecules and lipid transporters [17]–[20].

T3S effectors share a relatively unstructured, poorly conserved 20–30 amino acid signal sequence at their N-terminus that is often sufficient to mediate their broad secretion by T3S systems [21]. However, many effectors often require ancillary chaperone proteins for efficient translocation into target cells. Depending on the type of their client protein cargo, T3S chaperones can be divided into three classes (reviewed in [22]). Class III chaperones prevent pre-oligomerization of needle components in the bacterial cytoplasm before secretion; class II chaperones stabilize translocators by binding their hydrophobic regions, and class I chaperones stabilize and/or enhance effector secretion. Depending on the number of effectors with which they can associate, class I chaperones are further divided into class IA and IB [23]. Class IA chaperones are specific for single effectors and the genes encoding the chaperone-effector pair are often co-transcribed or adjacent to each other on the bacterial genome. Class IB chaperones associate with more than one effector and the genes encoding these chaperones are often unlinked from that of its cognate cargo. Many common features are shared by class I chaperones, including small size (15∼20 kDa) and acidic isoelectric points, a stable homodimer conformation, and ATP-independent chaperoning function [23]. Class I chaperones contain a basic core consisting of three α-helices and five β-sheets [24]; however, the degree of conservation at the amino acid level is limited, making the identification of new T3S chaperones difficult. From amino acid sequence analysis, *C. trachomatis* encodes at least six putative T3S chaperones: Slc1 (Ct043), Scc1 (Ct088), Scc2 (Ct576), Scc3 (Ct862), Ct274 and Scc4 (Ct663) [25], [26]. Several studies have validated their function as chaperones and have identified the substrates they engaged. For instance, Slc1 from *C. trachomatis* interacts with TARP and enhances its translocation/secretion in a heterologous *Yersinia* T3S system [27], [28]. Scc1 and Scc4 from *C. pneumoniae* enhance the secretion of CopN whereas Scc3 inhibits CopN secretion [29]. Additional T3S chaperones have been defined functionally. For example, Mcsc (Multiple Cargo Secretion Chaperone), which does not share any obvious sequence homology with known chaperones, was identified based on its ability to bind and stabilize the Inc proteins Cap1 and Ct618 [30].

**Figure 1:**
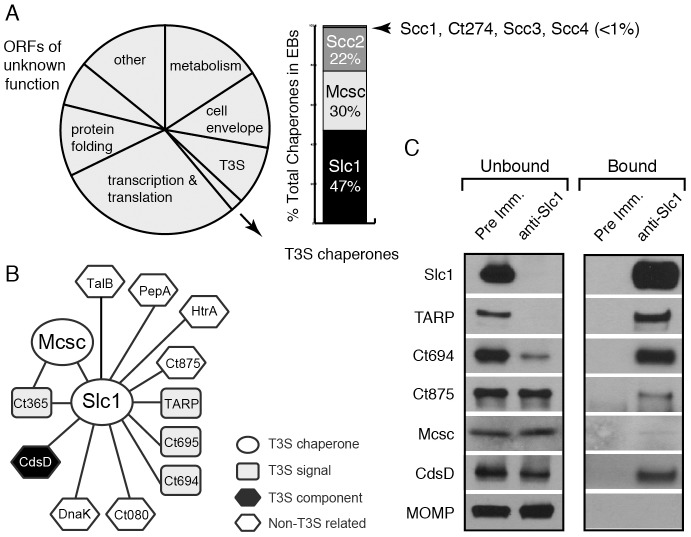
A distinct set of *Chlamydia* proteins associate with Slc1 in the Elementary Body (EB) form. **A**) Relative protein mass composition of the *C. trachomatis* EB. Approximately 2% of total EB protein mass is comprised of T3S chaperones, with Slc1, Scc2 and Mcsc accounting for over 99% of their mass. This figure represents a reanalysis of data reported in [31]. **B**) Protein interaction network for Slc1 and Mcsc. Cell lysates from gradient purified EBs were incubated with anti-Slc1 antibodies, anti-Mcsc antibodies, or non-specific IgG cross-linked to an agarose resin. Bound proteins were eluted at low pH, digested with trypsin and identified by liquid chromatography coupled to tandem mass spectrometry (LC-MS/MS) (Table S1). Only proteins that displayed specific interactions are shown. **C**) Specificity of Slc1-interactions. EB lysates were incubated with either anti-Slc1 antisera or pre-immune sera, and bound proteins were captured on Protein A/G agarose resin. The eluate (bound) and flow through (unbound) from both Slc1 IP and control IP were analyzed by immunoblotting with antibodies against selected proteins. MOMP is an abundant *Chlamydia* protein that serves as control for the specificity of the interactions shown.

A quantitative proteomic study indicated that the EB form of *C. trachomatis* is equipped with a complete set of T3 secretion components and is pre-packed with an abundant arsenal of putative T3S effectors and chaperones [31]. In addition to TARP, Ct694 and Ct695, over 50 *Chlamydia*-specific hypothetical proteins lacking signal peptides (∼7% of the molar mass of the EB form) were also identified. Since multiple effectors are likely translocated during invasion, a significant number of these *Chlamydia*-specific proteins may function as effectors with important roles early in infection. Interestingly, the EB form also contains a full complement of T3S chaperones, with Slc1 and Mcsc being the most abundant (Fig. 1A) [31]. Given their abundance and that genes encoding these chaperones are genetically unlinked from their effector protein cargos, we postulated that Slc1 and Mcsc may engage additional effectors in EBs.

In this study, we immunoprecipited Slc1 and Mcsc from *Chlamydia* EB lysates and identified co-purifying proteins by mass spectrometry. In this manner, we determined that Slc1 is in complex with multiple T3S effectors and that co-expression of Slc1 enhanced the secretion of these effectors in *Yersinia pestis*. In the process, we identified and characterized a new effector, TepP (Translocated early phosphoprotein – Ct875), which is tyrosine-phosphorylated upon translocation into host cells. Given that the majority of TARP, but not TepP, is pre-complexed with Slc1 within EBs, and that TepP phosphorylation at Tyr residues occurs later with respect to TARP phosphorylation, we postulate that Slc1 helps impart a hierarchy to effector translocation during *Chlamydia* entry. We further show that phosphorylated TepP associates with host scaffolding proteins Crk-I and Crk-II and that the recruitment of Crk proteins to nascent inclusions is absent in cells infected with a *Chlamydia* mutant harboring a *tepP* null allele and is restored in complemented strain. In addition, endocervical epithelial cells infected with this mutant exhibited transcriptional changes in a subset of innate immunity-related genes. We propose a model wherein TepP acts downstream of TARP to recruit scaffolding proteins that initiate and amplify signaling cascades important for establishing a replicative niche for *Chlamydia* within the infected host.

## Results

### The type III secretion chaperone Slc1 associates with multiple *C. trachomatis* proteins

A quantitative proteomics analysis of *C. trachomatis* indicated that around 2% of the EB total mass is composed of T3S chaperones, with Slc1, Mcsc and Scc2 accounting for over 99% of all known T3S chaperones (Fig. 1A) [31]. Slc1 forms complexes with TARP and enhances TARP translocation into HeLa cells when both proteins are co-expressed in *Yersinia enterocolitica* [27], [28]. In EBs, the majority of TARP is in complex with Slc1, and both TARP and Slc1 are present at similar molar concentrations [31]. However, since *slc1* is located ∼500 kb from *tarP*, it is unlikely that Slc1 constitutes a TARP-specific Class IA T3S chaperone. Furthermore, because putative T3S effectors in EBs are present at a 10 fold molar excess over the three major T3S chaperones [31], we hypothesized that chaperones like Slc1 and Mcsc must bind multiple effectors. We further hypothesized that these chaperones bind their cargos in a hierarchical manner and thus impart coherence to effector secretion. To test this premise we determined the compendium of EB proteins that associate with Slc1 and Mcsc. We immunoprecipitated (IP) Slc1 and Mcsc under native conditions from EB lysates and identified all proteins that associated with each individual chaperone by LC-MS/MS (liquid chromatography-tandem mass spectrometry). As previously reported [27], [31] TARP was one of the major proteins that co-purified with Slc1. In addition, two predicted T3S effectors, Ct694 and Ct695, and two hypothetical proteins, Ct365 and Ct875 co-purified with Slc1 (Fig. 1B). As described in the sections below, we renamed Ct875 as TepP to reflect the new functions defined in this study for this protein. CdsD, a T3S apparatus component, and proteins involved in metabolism were also specifically detected in the Slc1 IP samples (Table S1). Interestingly, both Mcsc and Slc1 mutually co-IP each other. Since these chaperones are not predicted to form heterodimers [27], this result suggests that Slc1 and Mcsc homodimers may co-chaperone the same cargo, presumably Ct365, which was detected in both IP samples. We validated the LC-MS/MS results by immunoblot analysis of the IP materials and immunodepleted flow-through samples with antibodies specific to Slc1 and the identified co-precipitating proteins (Fig. 1C). We detected TARP, Ct694, Mcsc, CdsD and Ct875/TepP in samples immunoprecipitated with anti-Slc1 antisera but not with pre-immune antisera. In contrast, MOMP, a very abundant EB protein, was not detected in the Slc1 IP samples, highlighting the specificity of the immunoisolations. As we had previously observed, immunodepletion of Slc1 from EB lysates led to a co-depletion of TARP [31]. Similarly, Ct694 was also substantially depleted, indicating that these two effectors within EBs are largely present in Sc1-containing complexes. The other co-purifying targets were not efficiently co-depleted which suggest that the complexes were either unstable or present in sub-stoichiometric amounts.

### Slc1 forms stable complexes with the effectors TARP, Ct694, Ct695 and Ct875/TepP

**Figure 2:**
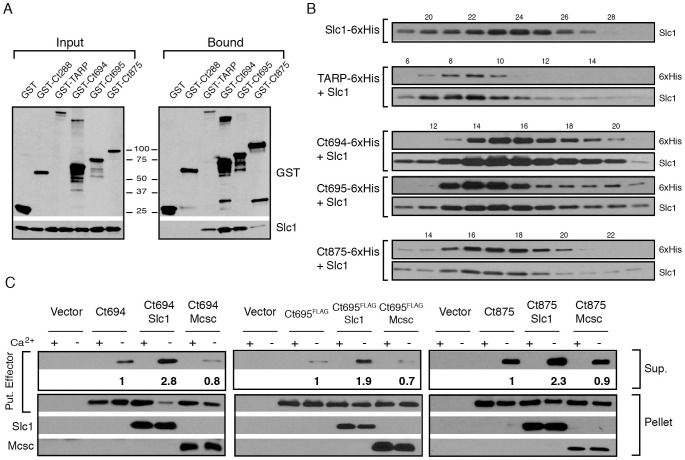
Slc1 associates as stable, multi-protein complexes with TARP, Ct694, Ct695 and Ct875/TepP, and enhances their secretion via the T3S system. **A–B)** Slc1 binds its putative target effectors *in vitro*. Slc1 was co-expressed in *E. coli* with GST-tagged TARP, Ct694, Ct695 and Ct875/TepP fusion proteins. The GST-tagged proteins were isolated from cell lysates with glutathione sepharose beads, and the relative levels of Slc1 co-isolated was assessed by immunoblot analysis (A). GST and GST-288 served as negative controls. To assess the relative size of these complexes, TARP, Ct694, Ct695 and Ct875/TepP were fused to a hexahistidine tag and co-expressed with Slc1. Bound proteins were purified using a Nickel resin, eluted with 500 mM Imidazole, and analyzed by gel filtration chromatography (B). Fraction numbers are provided on the top. Molecular size markers: Alcohol Dehydrogenase (150 kDa), Conalbumin (75 kDa) and Carbonic Anhydrase (29 kDa), peaked at F16-17, F20-21 and F26, respectively. No peak was observed between fractions 8–20 in Slc1-6xHis sample in the absence of co-expressed effectors. **C**) Slc1 enhances the T3S-dependent secretion of Ct694, Ct695 and Ct875/TepP. *Y. pestis* KIM8-E (Δ*ail*) was co-transformed with plasmids expressing Ct694, Ct875/TepP and FLAG-tagged Ct695 and untagged Slc1 or Mcsc in the combinations shown. T3S was induced by calcium chelation and the relative amount of protein secreted into the supernatants was assessed by quantitative immunoblots. Sup-cell free supernatant. Mcsc did not enhance the secretion of effectors, indicating that the secretion chaperone activity of Slc1 is specific for its target substrates.

We next tested if the major interactions identified for Slc1, especially those with potential T3S effectors, could be recapitulated *in vitro* with recombinant proteins. The most abundant proteins that associated with Slc1 are Ct875/TepP, TARP, Ct694, Ct695 and Ct365. We expressed these proteins as GST-tagged fusion proteins together with untagged Slc1 in *E. coli* and tested whether Slc1 would co-purify on glutathione sepharose beads. Because, we could not express the predicted inclusion membrane protein Ct365 [32] in *E. coli*, this potential effector was not studied further (data not shown). Pull downs of GST-tagged TARP, Ct694, Ct695 and Ct875/TepP with glutathione beads led to the co-isolation of Slc1. This binding was specific as neither GST nor GST-tagged Ct288, another inclusion membrane protein [33] that is present in EBs, led to Slc1 co-purification (Fig. 2A). T3S chaperones form stable complexes with a predicted 2∶1 stoichiometry of chaperone to effector protein [34]. To test if such larger complexes could form, we co-expressed in *E. coli* untagged Slc1 and hexahistidine-tagged full length TARP, Ct694, Ct695, or Ct875/TepP and isolated protein complexes on nickel resins. The bound material was eluted from the resin with imidazole and analyzed by gel filtration chromatography. All four proteins formed stable complexes with Slc1, with apparent molecular weights above 150 kDa, which are larger than that expected for 2∶1 chaperone effector complexes (Fig. 2B). Although gel filtration cannot always accurately predict molecular sizes for non-globular proteins [35], given that TARP forms hexamers and that the oligomerization domain is distinct from the Slc1 binding domain [14], [27], we postulate that size discrepancies between Slc1/TARP and the other Slc1/effector complexes represent the formation of higher order oligomeric forms.

### Slc1 enhances the secretion of Ct694, Ct695 and Ct875/TepP in a heterologous T3S system

Based on the gel filtration results we speculated that Slc1, as reported for TARP [27], would act as T3S chaperone and enhance the secretion of Ct694, Ct695, and Ct875/TepP. To test this premise, we reconstituted chaperone-assisted T3S in *Y. pestis* as previously described [29]. Effectors were expressed in *Y. pestis* as either untagged (Ct694 and Ct875) or FLAG-tagged (Ct695) proteins under the control of an arabinose-inducible promoter in the presence of Slc1 or Mcsc. Upon induction of T3S by calcium chelation, Ct694 and Ct695 were secreted into the supernatants as previously reported [15]. The secretion of Ct694 and Ct695 was enhanced two to three fold when Slc1, but not Mcsc, was co-expressed, suggesting that Slc1 functions as a *bona fide* T3S chaperone specific for these proteins (Fig. 2C). Consistent with the known behavior of most T3S chaperones, neither Slc1 nor Mcsc were secreted into supernatants (data not shown). The role of Slc1 in TARP secretion in this system could not be determined as expression of full length TARP in the presence of Slc1 hindered *Y. pestis* T3S (data not shown). Full length Ct875/TepP was also a target of secretion by the *Y. pestis* T3S system, even though Ct875/TepP is not predicted to have a T3S signal [21], [31], [36], and its secretion was enhanced by Slc1.

**Figure 3:**
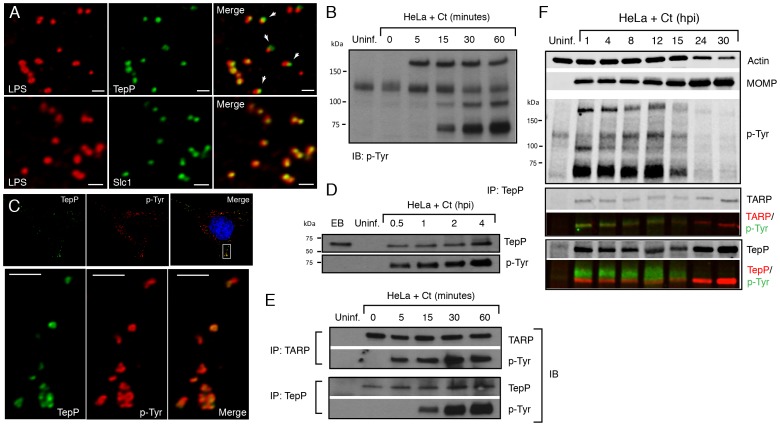
TepP/Ct875 is secreted at *Chlamydia* entry sites and is phosphorylated upon translocation. **A**) TepP is secreted from EBs early during infection. HeLa cells were infected with LGV-L2 at a MOI of 20 for 2 hr. Cells were fixed, permeabilized and immunostained with antibodies against *Chlamydia* LPS (red), Ct875/TepP (green) and Slc1 (green). Scale bar: 1 µm. **B**) Immunoblot analysis of total cell lysates of *Chlamydia* infected cells shows the appearance of at least three major tyrosine-phosphorylated proteins (>150 kDa, 100 kDa, and 65–70 kDa) within 1 hpi. **C**) TepP colocalizes with anti-phospho-Tyr signals at *Chlamydia* entry sites. HeLa cells were infected as in A) and immunostained with anti-TepP (green) and anti-p-Tyr (red) antibodies. Host and bacterial DNA were stained with DAPI (blue). Scale bar: 10 µm (upper panel); 2 µm (lower panel). **D**) TepP is phosphorylated at tyrosine residues upon EB association with host cells. Cells were infected as in B) with an MOI of 100 for the indicated times and TepP was immunoprecipitated from cell lysates and analyzed by immunoblotting with anti-TepP and anti p-Tyr antibodies. TepP was recognized by anti p-Tyr antibodies only upon association with host cells. **E–F**) TARP and TepP are tyrosine-phosphorylated at different rates during *Chlamydia* infection. HeLa cells were infected as in B) and total protein lysates were generated at 0, 5, 15, 30 min and 1 hpi and TepP and TARP was IP as above with anti-TARP or anti-TepP antibodies followed by immunoblot analysis with anti-TARP, -TepP and p-Tyr antibodies (E). Note relative delay in TepP tyrosine phosphorylation with respect to TARP. The prominent higher molecular weight tyrosine-phosphorylated band corresponds to TARP, as assessed by 2-color immunofluorescence-detection. In contrast, TepP only partially overlaps with the major 65–70 kDa tyrosine-phosphorylated proteins (F). MOMP: major outer membrane protein, Uninf: uninfected.

Ct875/TepP is the most abundant *Chlamydia*-specific hypothetical protein pre-packed in EBs [31]. We speculated that this protein is secreted early during EB attachment to cells and that it plays a role in invasion and/or establishment of the nascent inclusion. Consistent with this, an analysis of Ct875/TepP localization by immunofluorescence microscopy indicated a close association of Ct875/TepP with EBs by 2 hours post infection (hpi) in HeLa cells (Fig. 3A). However, the localization of Ct875/TepP was distinct from that of chlamydial LPS, suggesting translocation of Ct875/TepP from the EB at attachment sites (Fig. 3A). This localization pattern is similar to what has been described for TARP and Ct694 [15], and is clearly distinct from that of Slc1 which is mostly localized to the bacterial cytosol. Overall, these findings provide experimental evidence that Ct875/TepP is a new T3S effector translocated early during EB entry into epithelial cells.

### TepP (Ct875) is phosphorylated upon infection with kinetics distinct from TARP

Approximately 5–6 distinct proteins are tyrosine-phosphorylated early upon infection with *C. trachomatis* [37], [38]. Most of these proteins were originally assumed to be host proteins that were phosphorylated as a consequence of the activation of multiple kinase signaling pathways by *Chlamydia*, until TARP was identified as one of these major tyrosine-phosphorylated proteins [12]. Because Ct875/TepP has a molecular weight of 65 kDa, similar to one of the major tyrosine-phosphorylated protein detected within 15 minutes of *C. trachomatis* LGV-L2 infection [37] (Fig. 3B), we considered the possibility that Ct875/TepP is also tyrosine-phosphorylated. Consistent with this, analysis by indirect immunofluorescence microscopy indicated that anti-Ct875/TepP and anti-phosphotyrosine signals co-localized around bacteria at 2 hpi (Fig. 3C). Next, we determined that Ct875/TepP immunoprecipitated from infected HeLa cell lysates, but not from EBs lysates, was detected by anti-phosphotyrosine antibodies in immunoblots, suggesting that Ct875/TepP is tyrosine-phosphorylated upon association with host cells (Fig. 3D). Phosphorylation of TepP provided us an opportunity to monitor the kinetics of its translocation into cells. EB infections were synchronized at 4°C and shifted to 37°C for various time intervals within a 1 h span. Infected samples were lysed, split in two and TARP and TepP were immunoprecipitated. Total TARP, TepP and tyrosine-phosphorylated protein in the IP material was monitored with specific antibodies. We observed that tyrosine phosphorylation of TepP was delayed with respect to TARP by ∼10 min (Fig. 3E), suggesting either that kinases responsible for TepP phosphorylation are recruited with delayed kinetics to EB entry sites or that TARP translocation from EBs precedes TepP. However, TepP might not be the major ∼65–70 kDa phosphoprotein since the TepP band only partly overlapped with the major ∼65–70 kDa phosphotyrosine protein bands in dual labeling immunoblots (Fig. 3F).

**Figure 4:**
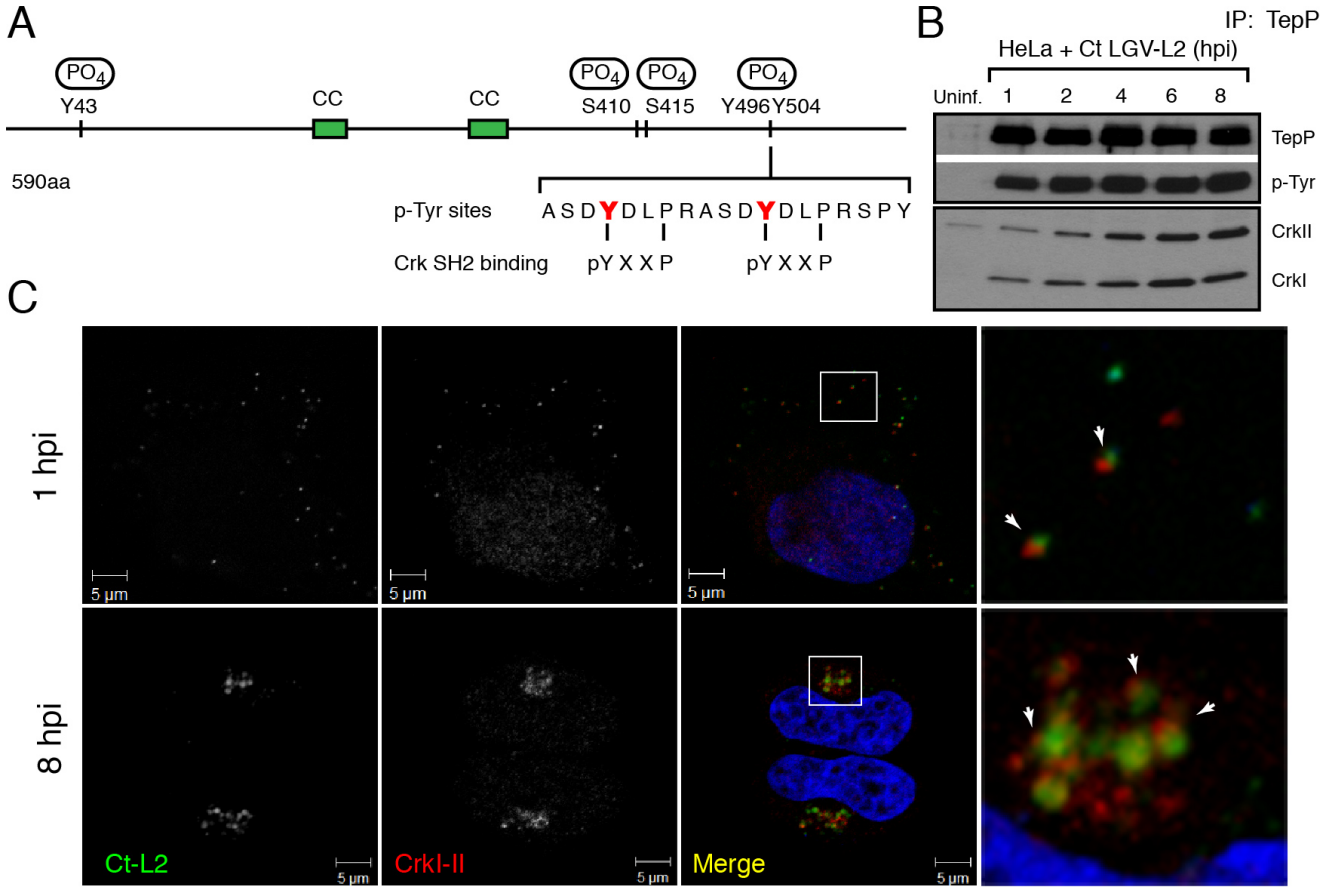
Crk-I and Crk-II bind to tyrosine-phosphorylated TepP and are recruited to nascent inclusions early during infection. **A**) Cartoon schematic of TepP phosphorylation sites identified by mass spectrometry (Fig. S1). **B**) CrkI and CrkII co-IP with TepP. HeLa cells were infected with L2 at MOI of 100 for 1, 2, 4, 6 and 8 hours. Cell lysates were immunoprecipitated with anti-TepP antibodies and the bound proteins were analyzed by immunoblot with anti-Crk, TepP and p-Tyr antibodies. Both isoforms of Crk, CrkI (lower band) and CrkII (upper band), co-IP with TepP. Uninf-uninfected. **C**) CrkI-II is recruited to *Chlamydia* entry sites and nascent inclusions. HeLa cells were infected with L2 at an MOI of 20 for 1 and 8 hours. Cells were fixed and stained with anti-MOMP (green) and anti-Crk (red) antibodies. Left panels are magnified images from boxed areas. Examples of sites of association between bacteria and Crk are marked by arrows. DAPI was used to stain nucleic acids. CrkI-II was recruited to *Chlamydia* entry sites by 1 hpi and this association continued after the nascent inclusions had trafficked to the host cells' perinuclear region.

We proceeded to map the phosphorylation sites on translocated Ct875/TepP by mass spectrometry. Infections were scaled up and Ct875/TepP was IP at 4 hpi in the presence of phosphatase inhibitors and processed for phospho-proteomics analysis. Four phospho-peptides were detected in the sample: two contained phosphorylated tyrosine (Y43 and Y496) and two phosphorylated serine residues (S410 and S415) (Fig. S1, Fig. 4A). Because the peptide spanning Y496 is part of a tandem repeat (ASD**Y**DLPR), it is unclear if one or both tyrosine residues (Y496 and Y504) are phosphorylated. Overall, these results indicate that Ct875/TepP is phosphorylated at multiple residues during infection and thus we renamed this protein as TepP for translocated early phosphoprotein.

### Crk, an adaptor protein, associates with TepP and is recruited to nascent inclusions

Protein tyrosine phosphorylation plays an important role in signal transduction by mediating the recruitment of proteins containing Src homology 2 (SH2) and/or phosphotyrosine binding (PTB) domains to target proteins [39]. Analysis of the TepP phosphopeptides using NetPhorest (http://netphorest.info/) [40] indicated the presence of a consensus pYxxP binding site (ASD**Y**DL**P**R) for the scaffolding protein Crk [41]. Crk is an adaptor protein that mediates phosphorylation-mediated regulation of cytoskeletal dynamics, cell adhesion and migration, phagocytosis and tumorogenesis [42]. Mammals express Crk-I and Crk-II, two alternatively spliced forms of *CRK*, and the Crk-like protein, Crk-L. Crk proteins contain SH2 and SH3 domains, which mediate binding to phosphorylated tyrosine residues and proline-rich domains, respectively [42]. To test if TepP translocated from EBs associated with Crk proteins, we performed a time course of infection and immunoprecipitated TepP in the presence of phosphatase inhibitors. *Chlamydia* infection did not alter the steady state levels of CrkI-II (Fig. S2); whereas increasing amounts of CrkI and CrkII co-IP with TepP as the infection progressed (Fig. 4B), indicating that these proteins are part of a complex. By immunofluorescence microscopy, we further observed endogenous Crk recruited to bacteria as early as 1 hpi and that this association continued past 8 hours, a point at which nascent inclusions have migrated to the microtubule organizing center (Fig. 4C). To test if the association of TepP with Crk is direct, we next examined if recombinant TepP could bind to Crk *in vitro*. We found that the binding between TepP and GST-Crk was significantly enhanced by *in vitro* phosphorylation of TepP and reduced after phosphatase treatment (Fig. S3, Text S1), suggesting that TepP phosphorylation is important for the recruitment of Crk. Together, these data are consistent with a model wherein TepP, after being secreted into host cells, is tyrosine-phosphorylated and recruits Crk to nascent inclusions, presumably through interactions with its SH2 domain.

### TepP is required for the recruitment of Crk to nascent inclusions

**Figure 5:**
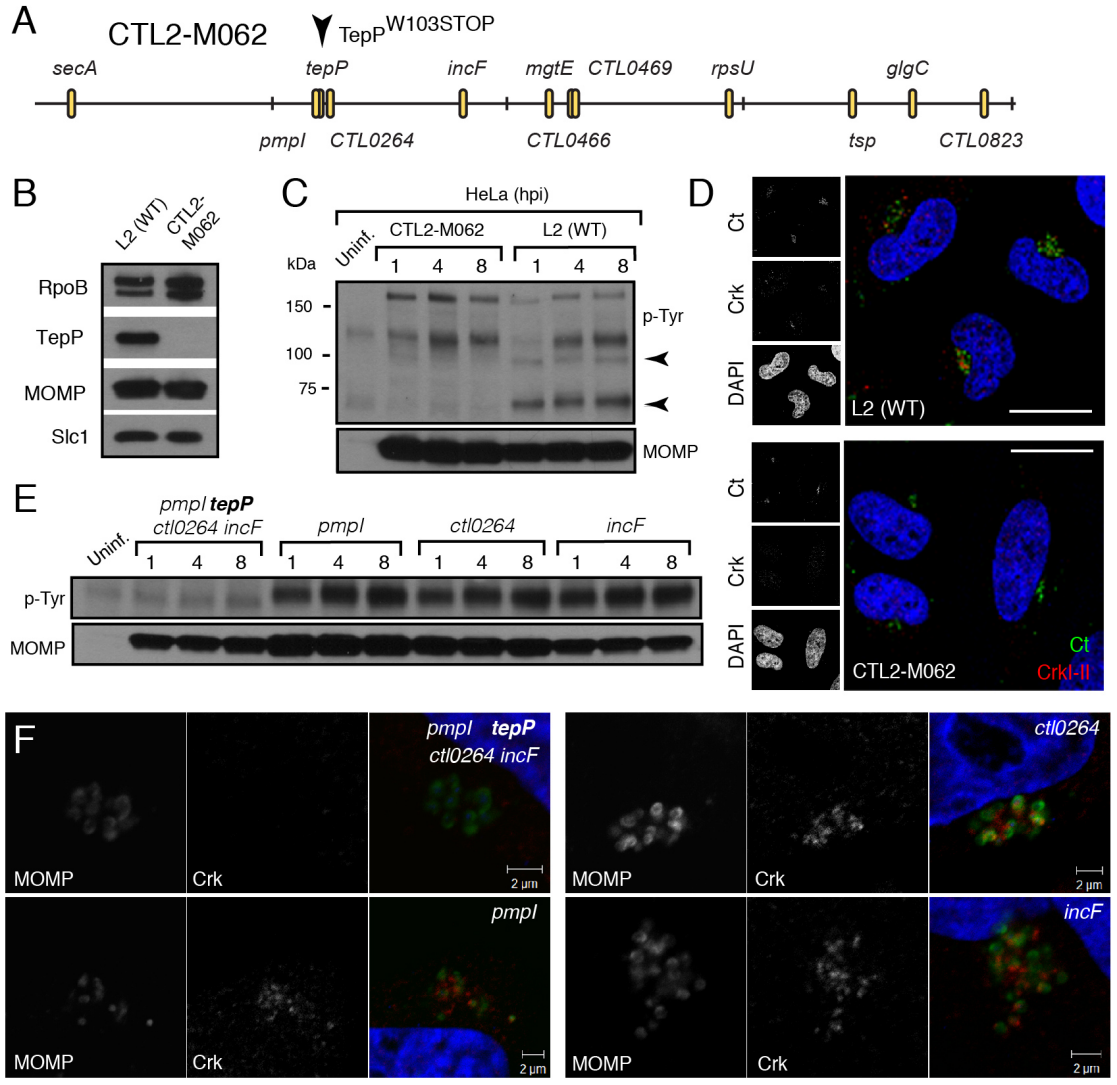
TepP is required for the recruitment of Crk to nascent inclusions. **A**) Physical map of single nucleotide variants identified in strain CTL2-M062, a chemically derived LGV-L2 mutant containing a nonsense mutation in the codon for amino acid 103 of TepP. Non synonymous mutations were identified by whole genome sequencing and verified by Sanger sequencing. Key differences from the parental reference strain are highlighted. **B–C**) TepP is required for the accumulation of major tyrosine-phosphorylated proteins in *Chlamydia* infected cells. Immunoblot analysis of the EB lysate of CTL2-M062 shows no detectable levels of TepP compared to wild type LGV-L2 (WT) (B). HeLa cells were infected with wild type LGV-L2 (WT) or CTL2-M062 at an MOI of 50 and samples were collected at indicated time points and analyzed by immunoblot with antibodies against p-Tyr and MOMP. Arrows indicate major TepP-dependent tyrosine-phosphorylated proteins (C). **D**) Crk does not associate with nascent inclusions in CTL2-M062 infected cells. Immunofluorescence staining of HeLa cells infected with wild type LGV-L2 and CTL2-M062 for 8 h. Cells were stained with anti-Crk (red) and anti-MOMP (green) antibodies, and with DAPI (blue). Crk recruitment was absent in a TepP deficient strain. Scale bar: 20 µm. **E–F**) The *tepP* mutation is genetically linked to the lack of the infection-induced p65-70 kDa tyrosine-phosphorylated proteins and the loss of Crk recruitment to nascent inclusions. Various recombinant strains generated from a cross between CTL2-M062 and wild type LGV-L2 were tested for TepP expression (Fig. S4, S5) and tested for the presence of the p65-70 kDa p-Tyr proteins (E) and recruitment of Crk to nascent inclusions (F).

Although *Chlamydia* spp. lacks a system for performing targeted gene disruptions, chemically-derived mutants can be readily generated and loss-of-function mutations can be identified by whole genome sequencing [43]. Using such an approach, our group has recently generated and sequenced a comprehensive collection of ethyl methyl sulfonate (EMS)-derived mutants of *C. trachomatis* LGV-L2 (unpublished results). We identified 6 strains with mutations in TepP, including strain CTL2-M062, bearing a G to A transversion that led to a premature stop codon at amino acid 103 (W103*) (Fig. 5A). By immunoblot analysis, EBs from CTL2-M062 lacked any detectable TepP, suggesting that the truncated form of TepP was either unstable or not properly expressed (Fig. 5B). Interestingly, even though TepP is not the predominant ∼65–70 kDa tyrosine-phosphorylated protein observed early during *C. trachomatis* infection, these phosphorylated species were no longer detected when cells were infected with the TepP-deficient CTL2-M062 mutant (Fig. 5C); whereas the levels of the phospho-TARP band (∼150 kDa) were unaltered. These results indicate that TepP may induce the phosphorylation of additional host and or secreted bacterial proteins at the early stages of infection. Next, we determined whether Crk association with nascent inclusions was dependent on TepP. HeLa cells were infected with either wild type or the TepP-deficient *C. trachomatis* mutant and the co-localization of bacteria with Crk was assessed by immunofluorescence microscopy. Crk was no longer associated with intracellular CTL2-M062 by 8 hpi (Fig. 5D).

The original TepP deficient isolate carried an additional 11 EMS-induced single nucleotide variants (SNVs) (Fig. 5A; Table S3). To exclude the possibility that these SNVs contributed to the lack of Crk recruitment to nascent inclusions, we generated recombinant strains between the TepP deficient isolate (generated in a Rif^R^ background) and a spectinomycin (Spc^R^) LGV-L2 strain by co-infection of cells as previously described [43]. Double drug resistant recombinants were plaque purified and screened by allele-specific PCR to determine the relative segregation of mutations present in the original TepP-deficient isolate. A total of 200 recombinants were screened and four co-isogenic strains were identified where various assortment of mutations were present (Fig. S5). The marked reduction of the ∼65–70 kDa signal in phosphotyrosine immunoblots was only observed in the strain lacking TepP (Fig. 5E). Similarly, Crk recruitment to nascent inclusions was only impaired in strains encoding the TepP^W103*^ variant (Fig. 5F), providing further genetic support to the notion that TepP is directly responsible for the recruitment of Crk to inclusions.

**Figure 6:**
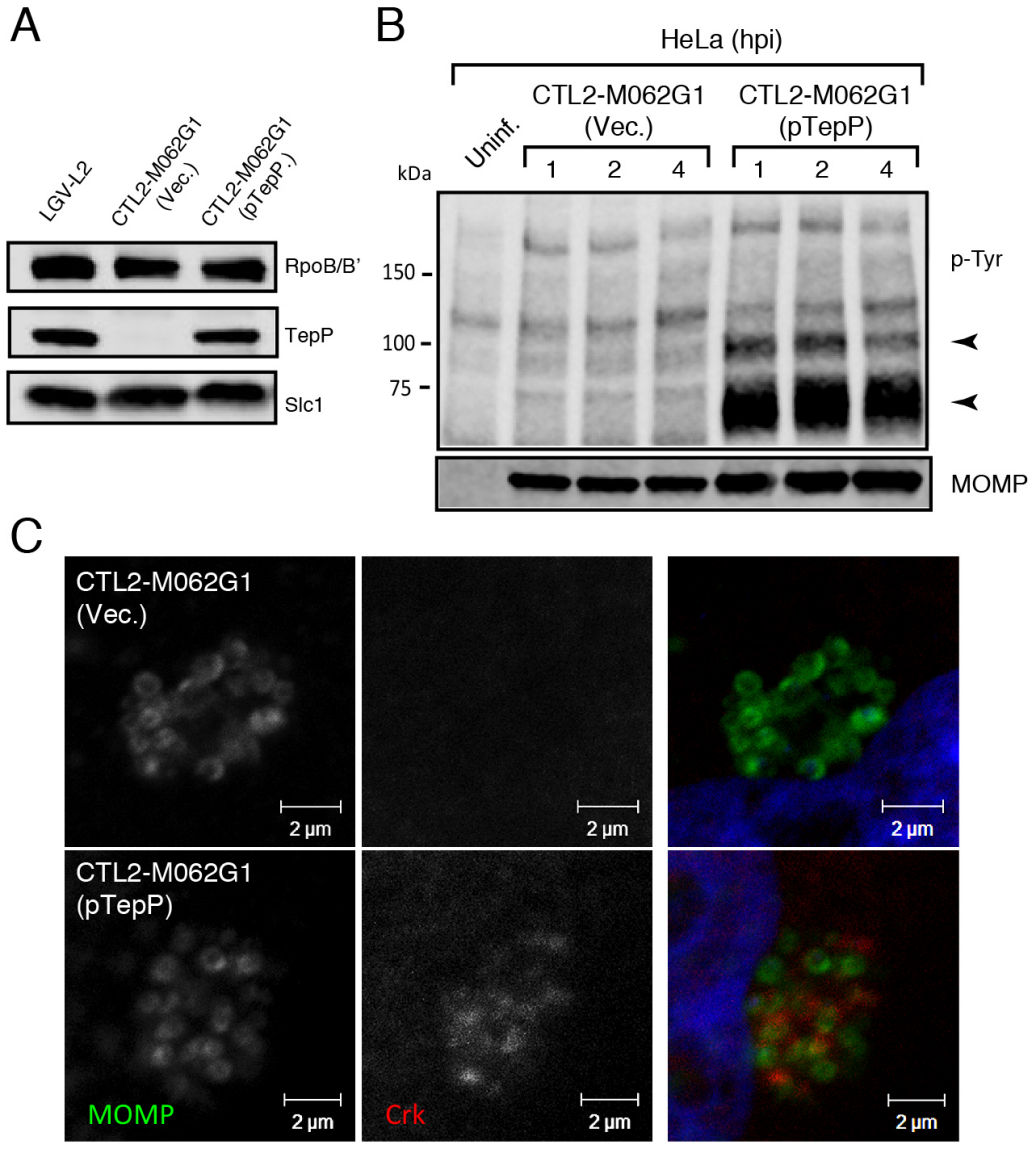
Genetic complementation of a *Chlamydia tepP* mutant restores the normal tyrosine-phosphorylation pattern of multiple proteins and rescues Crk recruitment to nascent inclusions. **A**) The recombinant TepP^W103*^ strain CTL2-M062G1 was transformed with an empty vector (Vec.) or a vector harboring the wild type *tepP* gene (pTepP). Immunoblot analysis of EBs derived from these strains with anti-TepP antibodies confirms the complementation of TepP expression to wild type levels (LGV-L2). Slc1 and RpoB/B' levels are shown as loading controls. **B**) The complemented recombinant TepP^W103*^ strain restored the pattern of tyrosine-phosphorylation induced during *Chlamydia* infection. Confluent HeLa cells were infected with CTL2-M062G1 strains described in (A) at an MOI of 50 and total protein lysates were collected at indicated time points. Samples were subjected to immunoblot analysis with antibodies against p-Tyr and MOMP. Arrows indicate phosphotyrosine bands restored after TepP complementation. **C**) The complemented recombinant TepP^W103*^ strains rescued Crk recruitment to nascent *Chlamydia* inclusions. Cells were infected for 8 hours with CTL2-M062G1 strains described in (A) at an MOI of 20, and immunostained with anti-Crk (red) and anti-MOMP (green) antibodies, and DAPI (blue).

Finally, to provide final genetic confirmation of the role of TepP in mediating these events, we took advantage of recent developments in DNA transformation in *C. trachomatis* [44]. We transformed the recombinant TepP^W103*^ strain with a *C. trachomatis-E. coli* shuttle plasmid expressing the *tepP* gene under the control of its endogenous promoter or the empty vector control and selected for stable transformants in the presence of penicillin. The transformed strains expressed TepP to a similar level as wild type strains (Fig. 6A). An immunoblot analysis of infected cells showed the restoration of the ∼65–70 kDa phosphotyrosine immunosignals in TepP complemented strains but not in strains transformed with the empty vector (Fig. 6B). In addition, Crk recruitment to nascent *Chlamydia* inclusions was restored (Fig. 6C). Both results, the restoration of protein phosphorylation and Crk recruitment to nascent inclusions, were observed when infecting A2EN cells, a newly derived endocervical epithelial cell line [45] (data not shown). Taken together, our results confirm that TepP is the major contributor, either directly or indirectly, to the tyrosine-phosphorylation of multiple proteins early during *C. trachomatis* infection and the subsequent recruitment of Crk to nascent inclusions.

### TepP regulates the expression of genes associated with innate immune responses

Although the CTL2-M062 had a ∼15 fold growth defect compared to the wild type parental strain, we could not unambiguously link this growth defect to strains bearing the *tepP* mutant allele (data not shown). To address whether TepP played a role in promoting *C. trachomatis* replication or infectivity, we compared the ability of the *tepP* mutants transformed with the *tepP* expressing plasmid or plasmid alone to generate infectious progeny. We observed no differences in the generation of EBs among these strains in either HeLa cells (Fig. S6D and Text S1) or in A2EN cells (data not shown). Similarly, silencing of Crk, a major TepP-binding partner, with siRNAs in HeLa cells did not impact *C. trachomatis* replication (Fig. S6A–C and Text S1).

**Figure 7:**
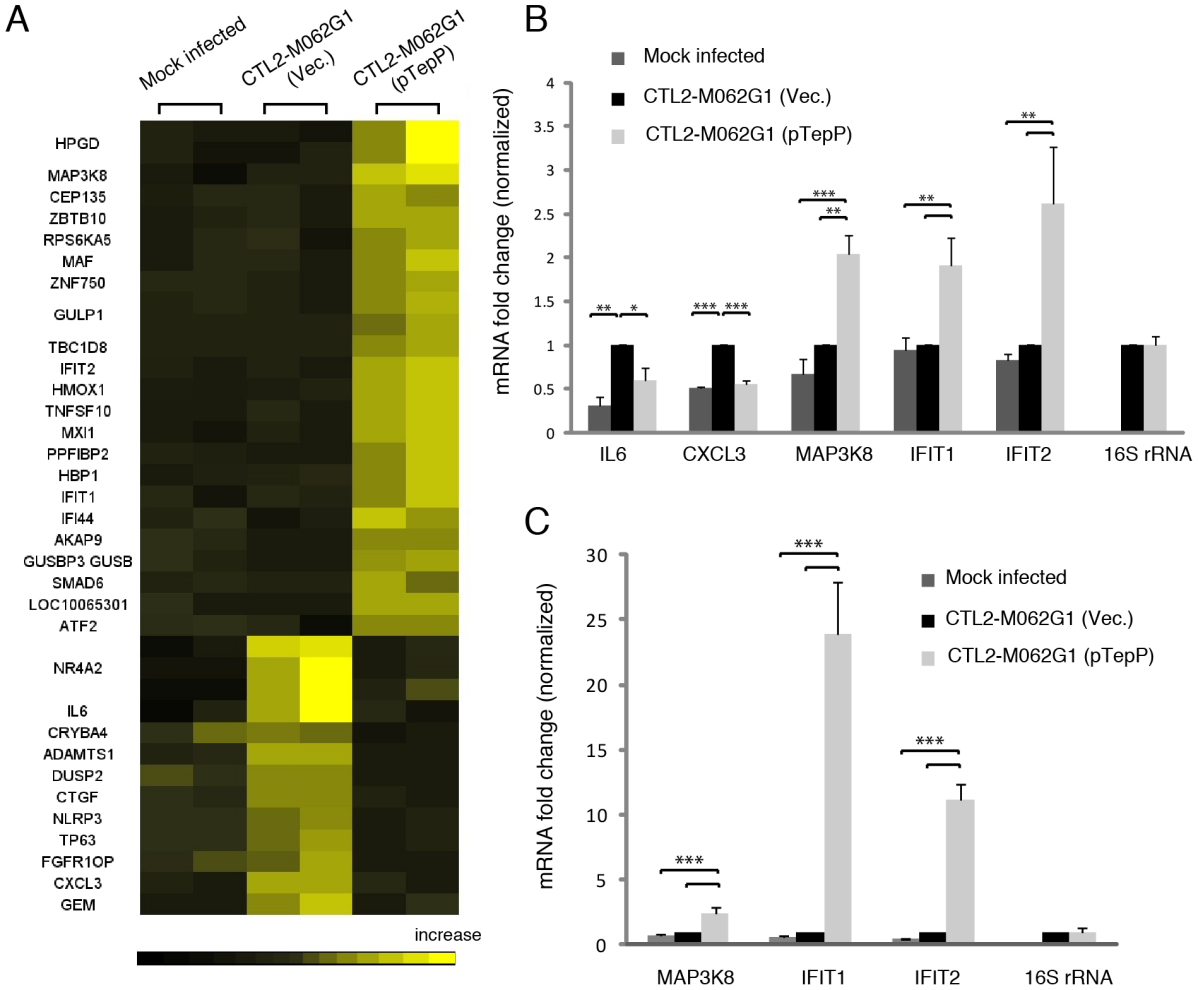
Global transcriptional profiling links TepP function to immune-related responses. **A**) Microarray analysis identified 33 host genes that have >1.5 fold-change in transcription between recombinant TepP^W103*^ strains (CTL2-M062G1) transformed with empty vector (Vec) and vector containing wild type *tepP* gene (pTepP). A2EN epithelial cells were infected for 4 hours. Results shown were from duplicate biological samples. **B**) Q-PCR results validated transcriptional change observed for 5 immune-related genes at 4 hpi. Fold-change in mRNA abundance was normalized to CTL2-M062G1 (Vec). **C**) TepP-dependent elevation of IFIT genes expression at 8 hpi. Q-PCR results of MAP3k8, IFIT1 and IFIT2 mRNA in A2EN cells infected as in (A). All data shown were as means ± standard deviations from three independent biological replicates. *, *P*<0.05; **, *P*<0.01; ***, *P*<0.001 (one-way analysis of variance and Tukey's multiple-comparison test).

These observations suggest that TepP is not essential for *C. trachomatis* replication in cell culture models of infection, although we cannot rule out a role for TepP in promoting invasion or intracellular survival in other cell types or in the context of intact tissues and an active immune system. However, given the observation that TepP recruits Crk, a scaffolding protein important in cell signaling, we hypothesized that the presence of TepP should lead to defined transcriptional responses by the infected cell. We compared the global transcriptional profile of mock infected A2EN cells or A2EN cells infected with the *tepP* mutant and its complemented counterpart for 4 h. A microarray analysis revealed 33 genes displaying greater than 1.5 fold changes in gene expression levels (Fig. 7A and Table S4). A Gene Ontology (GO) analysis of these differentially expressed genes revealed an enrichment for genes with immunity-related functions (data not shown). We next validated by quantitative-PCR, the expression of five of these genes: IL-6 and CXCL3, and MAP3k8, IFIT1 and IFIT2, whose transcription decrease and increase, respectively, depending on the presence of TepP (Fig. 7B). Interestingly, the fold-change for IFIT1 and IFIT2 changed from 2 fold to more than 10 fold by 8 hpi; whereas the level of fold-change for MAP3k8 did not (Fig. 7C). These data suggests that one of the functions of TepP is to modulate gene expression in the early stages of infection, presumably to impact the type and magnitude of the ensuing innate immune response.

## Discussion

Approximately 5–10% of the *C. trachomatis* genome encodes for proteins with putative T3S signals [21], [36]. These potential effector proteins are presumably translocated into host epithelial cells at various stages of infection to mediate epithelial cell invasion, establishment of a protected replicative vacuole, and evasion of innate immune responses (reviewed in [11]). Because *Chlamydia* cannot be readily manipulated with molecular genetic tools, most approaches to identify effectors have been indirect and relied on heterologous expression systems [46]. To date, only two effectors that are secreted in the early stage of infection, TARP and Ct694, have been experimentally validated in *C. trachomatis* [12], [15]. Given the complexity of interactions with the host cell during *Chlamydia* entry and nascent inclusion development, we expected additional effectors to be secreted upon the association of the EB with its target epithelial cell and that most of these effectors are pre-loaded in EBs. Indeed, a quantitative analysis of the *C. trachomatis* EB proteome [31] suggested the presence of at least 20 abundant hypothetical proteins with putative T3S signals. EBs are also pre-loaded with most of the predicted T3S chaperones encoded by the *Chlamydia* genome. However, putative T3S effectors are in molar excess to that of available T3S chaperones, which led us to speculate that these chaperones may engage multiple effectors.

The two most abundant T3S chaperones in EBs are the TARP chaperone Slc1 [27], [31] and Mcsc, which engages at least two inclusion membrane proteins during the RB stage [30]. Because the genes encoding Slc1 and Mcsc are unlinked from that of their effectors, we considered the possibility that these chaperones engage multiple effectors in EBs. To test this premise we decided to identify the set of proteins that stably associate with Slc1 and Mcsc at the EB stage by IP coupled to mass spectrometry. We only found a limited number of interactions for Mcsc in EBs. In contrast, Slc1 engaged at least four new substrates of the T3S system: Ct365, Ct694, Ct695 and Ct875/TepP, a protein not previously thought to harbor a T3S signal based on early prediction algorithms [21], [36]. Consistent with our findings, Mota's group recently showed that Slc1 can interact with Ct694 and Ct695 *in vitro* and enhances their secretion in *Y. enterocolitica* [28].

In addition to their traditional role in escorting cargo for secretion, T3S chaperones can regulate additional cellular functions. For instance, SicA, a *Salmonella* T3S chaperone for the effector SipA, directly interacts with the transcriptional activator InvF, and this complex activates the expression of T3S genes [47]. Similarly, SycD (LcrH), the chaperone for the translocator protein YopD, functions with YopD to represses Yop synthesis in *Yersinia pseudotuberculosis* [48]. In *Chlamydia*, the chaperone Scc4 (Ct663) negatively regulates σ^66^–dependent transcription by directly interacting with both σ^66^ and β subunits of RNA polymerase [49]. At least one T3S chaperone, the *Shigella* Spa15 protein, is secreted and plays a role in preventing apoptosis of the infected host cell [50]. Slc1 is unlikely to be a T3S cargo as it was not secreted by *Yersinia* (data not shown) and Slc1 localization was restricted to EBs early in infection (Fig. 3A). Nevertheless, we identified several additional proteins that specifically co-IP with Slc1, including the late transcription unit B protein (ltuB, Ct080), a serine protease (HtrA), a putative aminopeptidase (PepA) and a transaldolase (TalB). While not experimentally confirmed, it is unlikely that any of these proteins constitute T3S effectors as computational programs aimed at identifying T3S signals give these proteins very low confidence for T3S (www.effectors.org). At present, it is unclear whether these interactions are direct. However, one could envision scenarios wherein Slc1 could interact with non-effector proteins to signal the successful entry into a host cell after it is no longer physical bound to an effector. Such a mechanism might be especially important for an intracellular pathogen like *Chlamydia*, where physical engagement of the T3S apparatus may need to be coupled to rapid metabolic adaptations and post-translational modifications that drive successful colonization of the target cell.

**Figure 8:**
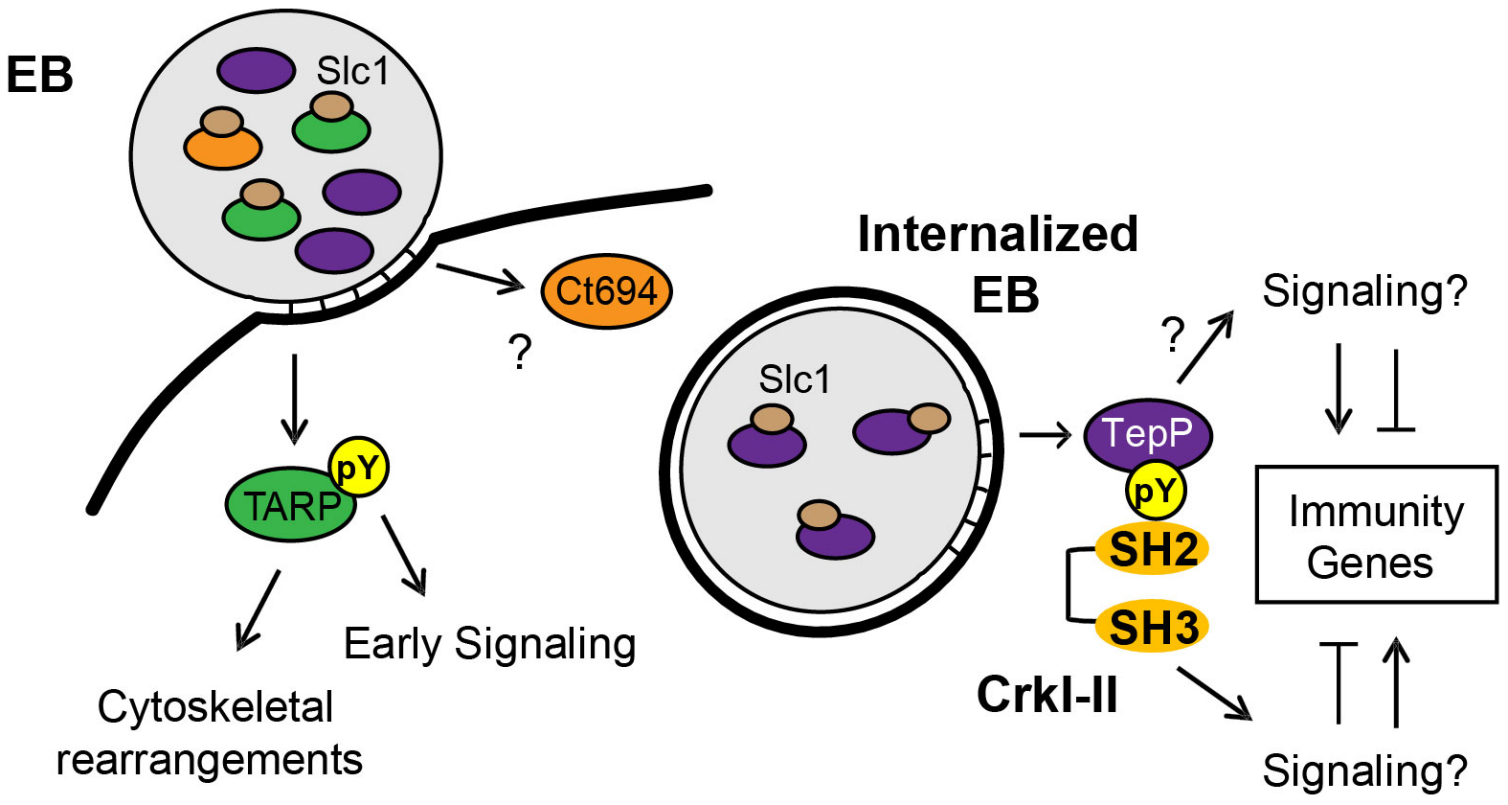
Model of the regulation and function of early effectors in signaling events occurring during the establishment of a nascent *Chlamydia* inclusion. The EB form of *C. trachomatis* is pre-loaded with multiple T3S effectors and chaperones. The majority of TARP and Ct694, two known early effectors, are pre-complexed with their cognate chaperone, Slc1, whereas only a small portion of TepP is in complex with Slc1. Upon *Chlamydia* attachment, TARP and Ct694 are translocated into host cell cytoplasm, freeing Slc1 to associate with TepP and enhance its secretion. TepP is translocated across membranes and phosphorylated by host tyrosine kinases. Phospho-TepP then recruits the host scaffolding protein, Crk, through interacting with the SH2 domain of Crk. The SH3 domain of Crk then recruits other host proteins to the nascent inclusion to initiate signaling cascades within the infected cell. TepP may also recruit additional proteins to initiate signaling responses independent of Crk.

T3S chaperones enhance the secretion of its bound cargo by stabilizing effectors in the bacterial cytoplasm and maintaining them in a secretion competent state [51]. In addition, T3S chaperones can also prioritize the secretion of effectors. For instance, YopE, a *Yersinia* T3S effector, lacking a SycE chaperone binding site, is secreted in the absence of other T3S effectors. However, YopE secretion is severely impaired when other effectors are allowed to compete with it, indicating that additional secretion signals are either unmasked or conferred by the T3S chaperone [52]. Similarly, CesT from enteropathogenic *Escherichia coli* (EPEC), binds to 9∼10 T3S effectors but one of the cargos, Tir, is secreted first and Tir secretion is important for secretion of the remaining effectors [53]. Indeed, a real-time analysis of CesT-dependent traslocation revealed a distinct order in the translocation of EPEC effectors and the effector-chaperone interaction is suggested to be one of the factors influencing translocation efficiency [54]. Based on these findings, we hypothesized that T3S chaperones would play a prominent role in establishing a hierarchy to the secretion of effectors by *Chlamydia* EBs. The most abundant T3S chaperone in EBs is Slc1, which, like CesT, engages multiple effectors. TARP, one of Slc1's cargos [27], is secreted within 5 min upon EB attachment to epithelial cells [12]. Interestingly, the majority of TARP in EBs is found pre-complexed with Slc1 (Fig. 1C) [31], implying that this pre-engagement with its chaperone could prime TARP for rapid secretion. Similarly, a significant proportion of Ct694 within EBs is pre-complexed with Slc1 (Fig. 1C), suggesting that Ct694 may be also secreted very early during infection, possibly at the same time as TARP. In contrast, only a minor portion of TepP was present in complexes with Slc1 in EBs, even though TepP is more abundant than TARP and Ct694 [31]. Given these observations, we considered a model wherein T3S substrates pre-bound by Slc1 in EBs, such as TARP and Ct694, will be delivered first, followed by TepP and potentially other effectors which do not exist as pre-formed effector-chaperone complexes in EBs. Consistent with this model, TepP was tyrosine-phosphorylated later than TARP (Fig. 3E). Although we cannot exclude the possibility that TepP-specific tyrosine kinases are recruited later than those that phosphorylate TARP, our preliminary results indicate that TepP is phosphorylated by kinases that also target TARP (data not shown), making this possibility less likely. Overall, the delayed phosphorylation of TepP, coupled with the relative abundance of Slc1-TARP as compared to Slc1-TepP complexes within EBs lead us to propose that Slc1 imparts a hierarchy to the translocation of effectors during *Chlamydia* invasion (Fig. 8).

Our findings indicate that TepP is a novel *C. trachomatis* effector that is targeted for tyrosine phosphorylation upon delivery into epithelial cells. This protein is highly conserved in all serovars of *C. trachomatis*, including ocular, genital and LGV strains. It shares 49% identity with TC_0268, ortholog in *C. muridarum* and less than 25% identity with potential orthologs in other *Chlamydiae* spp (http://www.uniprot.org/blast). TepP is phosphorylated at both tyrosine and serine residues (Fig. 4A), with a phosphotyrosine residue mapping to the peptide ASD**Y**DLPR, which is repeated in tandem between amino acids 496–504. This phosphorylation site matches the pYxxP consensus binding site for the host adaptor proteins Crk-I and Crk-II [41]. Indeed, we found that both Crk proteins co-IP with endogenous TepP during the early stages of *Chlamydia* infection (Fig. 4B). This interaction is likely to be direct and dependent on tyrosine phosphorylation since recombinant TepP only interacts with GST-CrK upon *in vitro* phosphorylation (Fig. S3). Consistent with these observations, CrkI-II associated with EBs at entry sites and nascent inclusions (Fig. 4C). Importantly, the lack of CrkI-II association with nascent inclusions in a *tepP* null mutant strain and restoration after complementation provided genetic confirmation that TepP is required for Crk recruitment.

CrkI-II are well-characterized scaffolding proteins that organize cytoskeletal rearrangement and signal transduction events [42]. The SH2 domain of Crk interacts with tyrosine-phosphorylated proteins. The SH3 domain, in turn, can interact with multiple proteins including guanine nucleotide exchange factors (GEFs) such as C3G, Sos1 and Dock180, and c-Abl and PI3K (p85 subunit), a protein and lipid kinase (reviewed in [42]). The manipulation of Crk function by bacterial effectors is not unprecedented. In *Helicobactor pylori*, CagA, a Type IV secretion effector, is tyrosine-phosphorylated upon translocation and interacts with Crk [55]. This interaction then triggers downstream signaling events of Crk, including SoS1/H-Ras/Raf1, C3G/Rap1/B-Raf and Dock180/ELMO pathway, resulting in CagA-specific cell responses such as epithelial cell scattering and cell-cell dissociation [55]. In *Pseudomonas aeruginosa*, exoenzyme T (ExoT) ADP-ribosylates CrkI and CrkII thus preventing the binding of the Crk SH2 domain to focal adhesion complex proteins, paxillin and p130*^cas^* [56]. This leads to uncoupling of integrin signaling, actin depolymerization, and potentially contributes to the anti-phagocytic activity of this opportunistic pathogen. The function of Crk in *C. trachomatis* infection is less clear. Because the Crk-binding partners Sos1 and Dock180 activate Rac1 [57], [58] and Rac1 activation is partially required for *C. trachomatis* invasion [59], it is possible that TepP, in addition to TARP [13], contributes to Rac1 activation at bacteria entry sites through its recruitment of Crk. However, since TepP is not essential for bacterial entry or establishment of the replicative vacuole within epithelial cells (Fig. S6D), we see a limited role for this effector in the activation of these pathways. Similarly, RNAi-mediated silencing of CrkI-II in HeLa cells did not affect the ability of bacteria to enter and replicate in these cells (Fig. S6A–C). These findings are in contrast to observations made in *Drosophila* S2 cells where Crk was identified as a potential host factor important for *C. muridarum* growth [60], and suggest possible differences in cell lines or *Chlamydia* strains used. At this stage we do not know the full compendium of proteins recruited to nascent inclusions via TepP and Crk-mediated protein scaffolding. However, it is clear from the number of proteins that are tyrosine-phosphorylated in a TepP-dependent manner that TepP contributes to multiple signaling events during *Chlamydia* invasion.

Since signaling events often lead to changes in gene transcription, we performed microarray analysis to compare the global transcriptional response of A2EN cells to infection with the *tepP* mutant versus its complemented counterpart. Interestingly, many immune-related genes showed a TepP-dependent activation. Among them, the most striking changes were observed for IFIT1 and IFIT2 with a fold-change more than 10 fold by 8 hpi (Fig. 7C). IFIT1 and IFIT2 belong to a family of interferon-induced protein with tetratricopeptide repeats (IFITs) that have a well-established role in host anti-viral defense. IFIT proteins repress the translation of viral genes by binding to eIF3, a translation initiation factor, and virus RNA bearing 5′-triphosphate, leading to suppression of virus replication [61]–[63]. In addition, ectopic overexpression of IFIT2 promotes cell death by a mitochondrial pathway, revealing another potential anti-viral mechanism for these proteins [64]. The role of IFIT proteins in bacterial infections is less clear. In murine macrophages, overexpression of IFIT2 represses lipopolysaccharide (LPS) induced TNF- α and IL-6 expression [65]. In contrast, IFIT2^−/−^ mice display reduced TNF- α and IL-6 in serum and LPS-mediated lethality in an endotoxic shock model, suggesting IFIT2 is a critical mediator for the secretion of LPS-induced proinflammatory cytokines [66]. In *C. trachomatis*, both IFIT1 and IFIT2 genes were reported to be up-regulated more than 10 fold in HeLa 229 cells by 16 hpi [67]. Our study shows that in the absence of TepP, the transcriptional level of IFIT1 and IFIT2 is similar to the level of uninfected A2EN cells, implying that TepP-mediated signaling regulates the expression of these genes during *C. trachomatis* infection. IFIT1 and IFIT2 can be induced directly by type I interferons [68], or indirectly after activation of host pattern recognition receptors [69]. We are currently investigating what TepP-mediated signal transduction pathways may mediate the expression of IFITs and other genes early in infection and the consequences of these transcriptional events in colonization of the host.

Taken together, our data suggests that TepP acts as a scaffolding protein that upon tyrosine phosphorylation, recruits additional scaffolding proteins like Crk, which in turn recruit more proteins to nascent inclusions, presumably to help establish an early niche for replication within the host (Fig. 8). In addition, the transcriptional response of host cells to infection with *tepP* mutants suggest that there is a distinct gene expression program that is dependent on TepP-mediated signaling events. We hypothesize that the compendium of genes activated and repressed in a TepP-dependent manner are important in establishing an immune environment within infected tissues that is more conducive to *C. trachomatis* colonization, survival and/or dissemination.

## Materials and Methods

### Cell lines, bacterial strains and reagents

*Chlamydia trachomatis* biovar LGV, serotype L2, strain 434/Bu was propagated in HeLa cells or Vero cells maintained in Dulbecco's Modified Eagle Medium (Sigma-Aldrich, St. Louis, Missouri, USA) supplemented with 10% fetal bovine serum (FBS) (Mediatech, Manassas, Virginia, USA). Human endocervical epithelial A2EN cells [45] were maintained in keratinocyte-SFM medium (Gibco, Life Technologies corp., Grand Island, NY, USA) supplemented with 10% FBS, 0.5 ng/mL human recombinant epidermal growth factor and 50 µg/mL bovine pituitary extract. EBs were purified by density gradient centrifugation using Omnipaque 350 (GE Healthcare, Princeton, New Jersey, USA) as previously described [31]. All recombinant protein expression was performed in *Escherichia coli* strain BL21(DE3). The *Y. pestis* KIM8-E (Δ*ail*) strain [70] used in this study is avirulent and is excluded from the National Select Agent Registry due to the lack of the 102-kb *pgm* locus [71]. In addition, this strain carries deletions of the *yopE*, *sycE* and *ail* genes and has been cured of the plasminogen activator (Pla)-encoding pPCP1 plasmid. All experiments with *Y. pestis* KIM8-E (Δ*ail*) were reviewed and approved by the Institutional Biosafety Committee at the University of Miami. All reagents used are of analytical grade.

### Generation of antibodies

Antibody generation was performed as previously described [30]. Briefly, recombinant proteins: His-tagged Slc1 (Ct043) and Mcsc (Ct260), and GST-tagged TepP (Ct875) were purified on affinity resins and used to immunize New Zealand White rabbits. Anti-GST antibodies were removed by pre-incubation with recombinant GST, and anti-TepP antibodies were affinity purified with TepP recombinant protein.

### Identification of proteins that interact with T3S chaperones

#### Immunoprecipitation (IP)

Antibodies against Slc1 and Mcsc were crosslinked separately to protein A/G resins (Pierce, Rockford, Illinois, USA) using 40 mM Dimethyl pimelimidate (Sigma-Aldrich, St. Louis, Missouri, USA) and the reactions were quenched with 40 mM ethanolamine (Sigma-Aldrich, St. Louis, Missouri, USA). Pre-immune serum was also crosslinked to resins and served as a negative control. Approximately 3×10^10^ EBs were lyzed by sonication in 1 mL Pierce IP lysis buffer (25 mM Tris, 150 mM NaCl, 1 mM EDTA, 1% NP-40, 5% glycerol; pH 7.4)(Pierce, Rockford, Illinois, USA) supplemented with 1 mM phenylmethylsulphonyl fluoride (PMSF) and 1X EDTA-free protease inhibitor cocktail (Roche, Basel, Switzerland). After centrifugation to pellet down insoluble debris, the lysate was incubated with the antibody-crosslinked resins at 4°C for 4 hours. After 3 washes with IP lysis buffer, the bound proteins were eluted with Pierce Elution buffer (pH 2.8) (Pierce, Rockford, Illinois, USA) and separated via SDS-PAGE on a 4–12% Bis/Tris gradient gel (Invitrogen Life Technologies, Carlsbad, California, USA). Lanes were sliced into 8–10 equivalent sections for in-gel digestion and analyzed by mass spectrometry at the Duke Proteomics Core facility.

#### Liquid chromatography electrospray ionization tandem mass spectrometry (LC-MS/MS)

The in-gel digested peptides from each band were analyzed via LC-MS/MS as follows: Approximately ½ of each digest (5 µL) was first trapped for 5 min on a 5 µm Symmetry C_18_ 180 µm I.D. ×20 mm column at 20 µL/min in 99.9% mobile phase A, then an analytical separation was performed using a 75 µm×250 mm BEH C18 column (Waters Corp., Milford, Maryland, USA) with a gradient of 5 to 40% mobile phase B over 30 minutes, with a flow rate of 0.4 µL/min at 55°C column temperature, using a nanoAcquity liquid chromatograph (Waters Corp., Milford, Maryland, USA). The mobile phase consisted of (A) 0.1% formic acid in water and (B) 0.1% formic acid in acetonitrile. Electrospray ionization was used to introduce the sample in real-time to a Synapt G2 Q-Tof mass spectrometer (Waters Corp., Milford, Maryland, USA), collecting data for each sample in data-dependent analysis mode with 0.6 second survey scans and three 0.6-second MS/MS scans in CID mode of the top three most abundant multiply-charged precursor ions. Raw data was processed in Mascot Distiller (v2.3) and searched in Mascot v2.2 (Matrix Science) against a concatenated database containing the deduplicated entries from NCBInr with *Chlamydia trachomatis* taxonomy (http://www.ncbi.nlm.nih.gov/pubmed/). For QToF data, search tolerances in Mascot were 10 ppm on precursor and 0.04 Da on product ions, requiring full trypsin specificity and allowing at most 2 missed cleavages. Carbamidomethylation (+57.0214 Da, Cys) was included as a fixed modification, and deamidation (Asn and Gln) and oxidation ((+15.9949 Da, Met) were allowed as variable modifications. Scaffold (v3.6.2, Proteome Software Inc.) was used to validate MS/MS based peptide and protein identifications. Peptide identifications were accepted if they could be established at greater than 80% probability as specified by the Peptide Prophet algorithm, and protein identifications were accepted if they could be established at greater than 90% Protein Prophet probability and contained at least 2 identified peptides [72], [73]. The overall peptide and protein false discovery rate is 0%. Results were from three independent biological replicates.

### Co-purification of Slc1 with GST-tagged effectors and gel filtration (size exclusion chromatography)

*E. coli* BL21(DE3) was co-transformed with a pET24d vector expressing Slc1 and pGEX vector alone or pGEX expressing individual GST-tagged test protein. Protein expression was induced using 0.5 mM isopropyl-1-thio-β-d-galactopyranoside (IPTG) for 3 hours at 37°C. Cells were pelleted and lysed by sonication in binding buffer (1% Triton X-100, 20 mM Tris, 150 mM NaCl, 1 mM EDTA, 1 mM PMSF, pH 7.4), and GST–tagged proteins were isolated from the supernatant using glutathione-Sepharose beads (GE Healthcare, Pittsburgh, PA, USA). After 4 h incubation, beads were washed 3 times with binding buffer, followed by 3 washes with washing buffer (binding buffer-0.2% Triton X-100, 300 mM NaCl). Bound proteins were solubilized in 1X Laemmli sample buffer (20 mM Tris-HCl, pH 6.8, 1% SDS, 5% Glycerol, 10 mM DTT, 0.01% bromophenol blue) and resolved by SDS PAGE, followed by immunoblot analysis with anti-Slc1 and –GST antibodies. Gel filtration chromatography was performed on proteins expressed in *E. coli* using a bi-cistronic vector to co-express untagged Slc1, with His-tagged test proteins. The protein complexes were first purified using a Nickel resin (GE Healthcare, Pittsburgh, PA, USA), eluted with 500 mM imidazole and applied to a Superdex 200 gel filtration column (GE Healthcare, Pittsburgh, PA, USA) for analysis. Fractions from each sample were collected and analyzed by immunoblot with anti-His and anti-Slc1 antibodies. Size markers, alcohol dehydrogenase (150 kDa), Conalbumin (75 kDa) and Carbonic Anhydrase (29 kDa) were purchased from GE and Sigma.

### *Yersinia pestis* T3S secretion assays

*Yersinia pestis* KIM8-E (Δ*ail*) was co-transformed with the compatible plasmids pBAD33 and pBAD24 (Table S2) either alone or in combination with pBAD plasmids encoding T3S chaperones and putative effectors. Transformed bacteria were grown overnight at 27°C in TMH media supplemented with ampicillin and chloramphenicol. Overnight cultures were used to inoculate fresh TMH media containing 2.5 mM calcium or without calcium at OD_620_ = 0.2 and incubated for 1 hour at 27°C. L-arabinose was added to 0.2% final concentration to induce chaperone and effector proteins expression and the culture temperature was shifted to 37°C for 5 hours. Bacteria were harvested by centrifugation and cell pellets were separated from culture supernatants. Proteins in the supernatant fractions were precipitated with 10% (v/v) trichloroacetic acid, resuspended in 1X SDS-PAGE sample buffer and normalized to the final OD_620_ of the respective culture.

### Indirect immunofluorescence staining

Approximately 5×10^4^ HeLa cells/well were seeded onto glass coverslips placed in a 24 well plate. The following day, cells were incubated with LGV-L2 EBs at an MOI of 20 and infections were synchronized by centrifugation (3000 rpm for 30 min) at 10°C followed by transferring the plates to a 37°C, 5% CO_2_ humidified incubator. At the indicated time points, the coverslips were fixed either with 100% methanol on ice for 15 min or with 3% formaldehyde/0.025% glutaraldehyde at room temperature for 20 min. Cells were then permeabilized with 0.2% Triton in phosphate buffer saline solution (PBS), blocked with 3% BSA in PBS for 30 min and stained with antibodies against TepP (1∶10), LPS (1∶250) (EV1-H1), MOMP (1∶250) (gift from K. A. fields), phosphotyrosine (1∶100) (Cell signaling #9411), Slc1 (1∶100), Crk (1∶10) (BD Transduction Laboratories 610035 clone 22). DAPI (Invitrogen Life Technologies, Carlsbad, California, USA) was used for staining nucleic acids. To permeabilize EBs at very early time points, 0.005% SDS in PBS was used. The images were further deconvolved using Huygens software (Scientific Volume Imaging, Hilversum, Netherlands).

### Protein lysates and IP of effector proteins

For short-term infections, ∼10^5^ HeLa cells were seeded per well in 24-well plates the day before experiment. Cells were incubated with LGV-L2 or its mutant derivatives at a MOI of 100 and infections were synchronized by centrifuging at 3000 rpm for 30 min at 10°C, followed by a shift to 37°C. Samples were collected at indicated time points by washing the well once with PBS followed by adding 90 µL 2X Laemmli sample buffer. For IPs of effector proteins, ∼5×10^6^ HeLa cells were pre-seeded into a 10-cm cell culture dish the day before infections and cells were incubated with LGV-L2 or its mutant derivatives at a MOI of 100. Infections were synchronized by pre-incubation for 1 hour at 4°C in Hanks balanced salt solution, followed by the addition of DMEM media pre-warmed to 37°C. At the indicated time points, infected cells were washed two times with ice-cold PBS and lyzed in 1 mL Pierce IP lysis buffer supplemented with 1 mM PMSF, protease inhibitor cocktail and Halt phosphatase inhibitor (Pierce, Rockford, Illinois, USA). After sonication and high speed centrifugation to remove insoluble debris, antibodies against TARP [74] or TepP were added to cell lysates and incubated for 3 hours at 4°C. Protein A resin was added to isolate immunocomplexes. After washing thoroughly with Pierce IP lysis buffer, bound proteins were resolved by SDS PAGE and subjected to immunoblot analysis.

### Identification of TepP phosphorylation sites by LC-MS/MS

**IP.** Two 15-cm cell culture dishes of confluent HeLa cells were infected with LGVL-2 at a MOI of 100 as described above. Another two dishes of confluent HeLa cells were mock-infected. Four hours post treatment, cells were collected, washed twice with PBS and lyzed as previously described [75] to generate cytosolic and nuclear/EB fraction. After clarifying the cytosolic fraction by high speed centrifugation, antibodies against TepP were added to the infected and control cell lysates and incubated for 3 hours at 4°C with continuous mixing. Immunocomplexes were purified with protein A resin and bound proteins were solubilized in SDS sample buffer.

#### Phosphopeptide sample preparation and nano-flow LC-MS/MS analysis

Following 1D-SDS-PAGE separation, the molecular weight region corresponding to that of TepP was excised and subjected to an in-gel trypsin digestion as previously described [76]. Extracted peptides were brought to dryness using vacuum centrifugation and resuspended in 100 µL 80% acetonitrile, 1% TFA, 50 mg/mL MassPrep Enhancer, pH 2.5 (Waters Corp., Milford, Maryland, USA). Phosphopeptides were enriched using a 200 µL TiO2 Protea Tip (Protea Bio., Morgantown, WV, USA) and subsequently washed with 200 µL 80% acetonitrile, 1% TFA, 50 mg/mL MassPrep Enhancer followed by 200 µL 80% acetonitrile, 1% TFA. Peptides were eluted in 50 µL 20% acetonitrile, 5% aqueous ammonia, pH 10.5 and then acidified to pH 2.5 with formic acid prior to drying using vacuum centrifugation.

Samples were resuspended in 10 µL 2% acetonitrile, 0.1% formic acid, 10 mM citric acid and subjected to chromatographic separation on a Waters NanoAquity UPLC, in the same manner as described for the immunoprecipitation samples, with the following exceptions: the analytical column was held at 5% B for 5 min at the beginning of the analytical separation, prior to a linear elution gradient of 5% B to 40% B over 30 min. The analytical column was connected to a fused silica PicoTip emitter (New Objective, Cambridge, MA) with a 10 µm tip orifice and coupled to an LTQ-Orbitrap XL mass spectrometer through an electrospray interface. The instrument was set to acquire a precursor MS scan in the Orbitrap from *m/z* 400–2000 with r = 60,000 at *m/z* 400 and a target AGC setting of 1e6 ions. In a data-dependent mode of acquisition, MS/MS spectra of the three most abundant precursor ions were acquired in the linear ion trap with a target AGC setting of 5e3 ions. Max fill times were set to 1000 ms for full MS scans and 250 ms for MS/MS scans with minimum MS/MS triggering thresholds of 5000 counts. For all experiments, fragmentation occurred in the LTQ linear ion trap with a collision-induced dissociation (CID) energy setting of 35% and a dynamic exclusion of 60 s was employed for previously fragmented precursor ions.

#### Qualitative identifications and residue specific phosphorylation localization from raw LC-MS/MS data

Raw data was processed in Mascot Distiller (v2.3) and searched in Mascot v2.2 (Matrix Science) against a concatenated database containing the deduplicated entries from NCBInr with *Chlamydia trachomatis* taxonomy. Search tolerances for LTQ-Orbitrap XL data were 10 ppm for precursor ions and 0.8 Da for product ions using trypsin specificity with up to two missed cleavages. Carbamidomethylation (+57.0214 Da, C) was set as a fixed modification, whereas oxidation (+15.9949 Da, M) and phosphorylation (+79.9663 Da, S, T, and Y) were considered a variable modifications. All searched spectra were imported into Scaffold and confidence thresholds were set using the PeptideProphet and ProteinProphet algorithms which yielded a peptide and a protein false discovery rate of 0% [72], [73]. Phosphorylation site localization was assessed by exporting peak lists directly from Scaffold into the online AScore algorithm (*ascore.med.harvard.ed*) [77].

### Identification and whole genome sequencing of a TepP-deficient LGV-L2 strain

A strain containing a *G309A* (W103*) null allele in *tepP* was initially identified by whole genome sequencing of a collection of ethyl methyl sulfonate (EMS)-mutagenized and plaque-purified *C. trachomatis* LGV-L2 strains generated as previously described [43] (Bastidas R. J. and Valdivia R. H. unpublished results). Strain CTL2-M062 harboring *tepP G309A* was identified from a pool of 20 mutants by Sanger sequencing of the *tepP* locus (CTL0255). CTL2-MO62 was recovered in sucrose-phosphate-glutamate (SPG) buffer (0.22 M sucrose, 0.01 M potassium phosphate, 0.005 M L-glutamic acid, pH 7.0) after hypotonic lysis of a monolayer of infected HeLa cells grown in a 10-cm^2^ cell culture dish. Lysates were sonicated (2×10 seconds in ice water) and bacterial cells were spun down at 14,000 rpm, for 15 minutes at 4°C, and resuspended in 1X DNAse I buffer (New England Biolabs, Ipswich, MA, USA). Cell suspensions were treated with 4 Units of DNAse 1 (New England Biolabs, Ipswich, MA, USA) for 1 hour at 37°C to deplete contaminating host DNA. Following a wash with PBS buffer, total DNA was isolated with a DNA isolation kit (DNeasy tissue and blood kit, Qiagen, Valencia, CA, USA) as described by the manufacturer.

For whole genome sequencing, 1 µg of CTL2-M062 enriched DNA was fragmented with an Adaptive Focused Acoustics S220 instrument (Covaris, Inc. Woburn, MA, USA), and DNA sequencing libraries were prepared with a library construction kit (TruSeq DNA Sample Preparation Kit v2, Illumina, Inc. San Diego, CA, USA) according to the manufacturer's instructions. Libraries were sequenced in a MiSeq DNA Sequencing Platform (Illumina, Inc. San Diego, CA, USA) at the Duke University IGSP DNA Sequencing Core facility. Genome assembly and single nucleotide variant (SNV) identification was performed with Geneious Software version 6 (Biomatters - http://www.geneious.com/). The *C. trachomatis* L2 434/Bu genome (GenBank no. NC_010287) was used as reference sequence. All non-synonymous SNVs identified in CTL2-M062 (see Table S3) were independently verified by Sanger sequencing.

### Generation of *Chlamydia* recombinant strains

*Chlamydia* recombinants were generated as previously described [43]. Briefly, confluent Vero cells grown on a 24-well plate were co-infected with CTL2-MO62 (Rifampin resistant, Rif^R^) and a Spectinomycin resistant mapping strain (Spc^R^) at a ratio of 2∶4 and recombinant progenies were selected from among plaques that formed in the presence of Rif (200 ng/mL) and Spc (200 µg/mL). Plaque-purified recombinants were further expanded in Vero cells and genotyped with SNV specific primers.

### *Chlamydia* transformation

The gene encoding TepP and its predicted promoter region (300 bp upstream of the TepP start codon) was amplified by PCR from LGV-L2 genomic DNA and inserted into the *E. coli-Chlamydia* shuttle vector p2TK2-SW2 [78]. The recombinant TepP^W103*^ strains (G1) were transformed with either empty vector or vector harboring wild type TepP as previously described with some modifications [44]. Briefly, around 4×10^6^ Vero cells treated with transformation buffer (10 mM Tris pH 7.4 in 50 mM CaCl_2_) were infected with G1 pre-incubated with >6 µg plasmid in transformation buffer. Transformed *Chlamydia* was selected under 1U of penicillin G (Sigma P3032) for several passages. After initial selections, transformed *Chlamydia* was maintained in the presence of 10U penicillin G. TepP expression was confirmed by immunoblot analysis.

### Microarray analysis

Approximately 0.8×10^6^ A2EN cells were seeded per well in 6-well plates the day before experiment. Duplicate cell samples were mock infected, or infected with the *tepP* mutant strain transformed with empty vector or the vector harboring wild type *tepP* at an MOI of 10. Infections were synchronized by centrifugation at 3000 rpm for 30 min at 10°C, followed by an immediate shift to 37°C with pre-warmed cell culture media. Samples were collected at 4 hpi using QIAGEN RNeasy Plus Mini Kit (QIAGEN, Valencia, CA, USA) as described by the manufacturer. RNA integrity was assessed with Agilent 2100 Bioanalyzer G2939A (Agilent Technologies, Santa Clara, CA, USA) and quantified with a Nanodrop 8000 spectrophotometer (Thermo Scientific/Nanodrop, Wilmington, DE, USA). Hybridization targets were prepared with MessageAmp Premier RNA Amplification Kit (Applied Biosystems/Ambion, Austin, TX, USA) from total RNA, hybridized to GeneChip Human Genome U133A 2.0 arrays in Affymetrix GeneChip hybridization oven 645, washed in Affymetrix GeneChip Fluidics Station 450 and scanned with Affymetrix GeneChip Scanner 7G according to standard Affymetrix GeneChip Hybridization, Wash, and Stain protocols (Affymetrix, Santa Clara, CA, USA). Data analysis was performed using Partek Genomic Suite 6.6 (Partek Inc., Saint Louis, MO, USA).

### Quantitative real-time PCR (Q-PCR)

Q-PCR was performed using *Power* SYBR Green RNA-to-C_T_ 1-*Step* Kit and StepOne Real-Time PCR system as described by the manufacturer (Applied Biosystems, Grand Island, NY, USA). Primers specific for IL-6, CXCL3, MAP3k8, IFIT1, IFIT2, and Actin were designed using Roche Universal Probe Library (http://www.roche-applied-science.com) and are listed in Table S5. Primers against *Chlamydia* 16S rRNA were used to quantify the amount of bacteria in each sample (Table S5).

### Ethics statement

The use of rabbits for the generation of antisera (Protocol A301-11-12) was approved by the Duke University Office of Animal Welfare Assurance (OAWA) after review by the IACUC committee. The IACUC ensures compliance of this protocol with the U.S Animal Welfare Act, Guide for Care and Use of Laboratory Animals and Public Health Service Policy on Humane Care and Use of Laboratory Animals.

## Supporting Information

Figure S1 **M/Z spectra of TepP phosphopeptides.** Endogenous TepP was immunoprecipitated at 4 hpi. The corresponding TepP band was excised from the gel, digested with trypsin and phosphopeptides were enriched on a titanium dioxide affinity column, prior to elution and analysis by LC-MS/MS. Four phospho-peptides were detected: two phosphoserine and two phosphotyrosine. Detected peptides were shown above each spectrum and detected phosphorylation site was underlined. Y-axis is the relative intensity of peaks. X-axis is mass-to-charge ratio (m/z).
(TIF)

Figure S2 **Crk levels remain constant throughout infection.** Confluent monolayer of HeLa cells were infected with wild type LGV-L2 at an MOI of 50 and collected at indicated time points. Samples were subjected to immunoblot analysis with antibodies against Crk, Actin and MOMP. C: control uninfected cells.
(TIF)

Figure S3 **Recombinant TepP interacts with GST-Crk in a phosphorylation-dependent manner.** Purified GST or GST-Crk was incubated with purified TepP-6xHis or purified TepP-6xHis that had been phosphorylated *in vitro*. One sample of phosphorylated TepP-6xHis was treated with Calf intestinal Alkaline Phosphatase (PPase). The efficiency of TepP co-precipitation with GST-Crk increased after *in vitro* phosphorylation. Dephosphorylation with PPase decreased the efficiency of GST-Crk co-precipitation.
(TIF)

Figure S4 ***C. trachomatis*** **recombinant harboring a** ***tepP*** **null allele (** ***tepP*** W103*) does not express full length TepP. EBs of four recombinants were selected from a co-infection setting between CTL2-M062 (Rif^r^) and a Spc^r^ LGV-L2 variant. Recombinants were isolated by plaquing on Vero cell monolayers in the presence of rifampin and spectinomycin. Individual plaques were amplified on Vero cells, and EBs from recombinants with the genotypes shown (also see Fig. S5) were harvested and purified on density gradients. EBs were lyzed in SDS sample buffer, and subjected to immunoblot analysis with antibodies against TEPP and RpoB/B', MOMP and Slc1.
(TIF)

Figure S5 **Genotype of recombinant strains harboring products of a CTL2-M062 (Rif^r^) and a LGV-L2 Spc^r^ co-infection.** Black shading indicates single nucleotide variants (SNV) present in CTL2-M062. Each SNV was verified by Sanger sequencing.
(TIF)

Figure S6 **Replication potential of LGV-L2 in Crk knockdown cells and** ***tepP*** **mutants in epithelial cells.** A) Transfection of Crk siRNAs decreased the expression level of both CrkI and CrkII in HeLa cells. Upper panel is the immunoblot analysis of HeLa cells transfected with two different Crk siRNAs (3 and 5) or negative control siRNA (NC) for 48 h. Total cell lysates were probed with anti-Crk and anti-Nup62 (loading control) antibodies. B) Quantification of siRNA-mediated decreased levels of Crk protein expression as assessed by quantitative immunoblots on a LI-COR imager. CrkI and CrkII expression level was decreased around 50% after siRNA treatment. C) Crk siRNA knockdown does not affect *C. trachomatis* growth as assessed by IFU assay. IFUs were normalized to growth in cells treated with control siRNA (NC). D) Comparison of IFU burst between the *tepP* mutant CTL2-MO62G1, and its derivatives transformed with empty vector or pTepP. HeLa cells were infected for 28 h at an MOI<1. The resulting infectious progeny were titered in HeLa cells as described in Supplemental Material and Methods, and normalized to input number of bacteria. All data shown were as means ± standard deviations from experiments performed in triplicate.
(TIF)

Table S1 **Number of unique spectra identified by LC-MS/MS from samples immunoprecipitated with anti-Slc1 and anti-Mcsc antibodies from EBs.**
(DOCX)

Table S2 **Plasmid constructs used in this study.**
(DOCX)

Table S3 **Single nucleotide variants in strain CTL2-M062.**
(DOCX)

Table S4 **List of genes that display a TepP-dependent regulation at 4 hpi as determined by microarray analysis.**
(DOCX)

Table S5 **Primer sequences used for Q-PCR.**
(DOCX)

Text S1 **Supporting materials and methods.**
(DOCX)
