# Correction: The *Chlamydia trachomatis* Type III Secretion Chaperone Slc1 Engages Multiple Early Effectors, Including TepP, a Tyrosine-phosphorylated Protein Required for the Recruitment of CrkI-II to Nascent Inclusions and Innate Immune Signaling

**DOI:** 10.1371/journal.ppat.1004094

**Published:** 2014-03-26

**Authors:** The PLOS Pathogens Staff

There is an incorrect term used in a sentence in the Results section under the header “TepP is required for the recruitment of Crk to nascent inclusions.” The correct sentence is: We identified 6 strains with mutations in TepP, including strain CTL2-M062, bearing a G to A transition that led to a premature stop codon at amino acid 103 (W103*).

## References

[1] Chen Y-S, Bastidas RJ, Saka HA, Carpenter VK, Richards KL, et al. (2014) The *Chlamydia trachomatis* Type III Secretion Chaperone Slc1 Engages Multiple Early Effectors, Including TepP, a Tyrosine-phosphorylated Protein Required for the Recruitment of CrkI-II to Nascent Inclusions and Innate Immune Signaling. PLoS Pathog 10(2): e1003954 doi: 10.1371/journal.ppat.1003954 24586162 10.1371/journal.ppat.1003954 PMC3930595

