# Supplementary figures and images for: The Chlamydia trachomatis Type III Secretion Chaperone Slc1 Engages Multiple Early Effectors, Including TepP, a Tyrosine-phosphorylated Protein Required for the Recruitment of CrkI-II to Nascent Inclusions and Innate Immune Signaling
Source: PLoS Pathog. 2014 Feb 20;10(2):e1003954. doi: 10.1371/journal.ppat.1003954 (PMC3930595; doi:10.1371/journal.ppat.1003954)

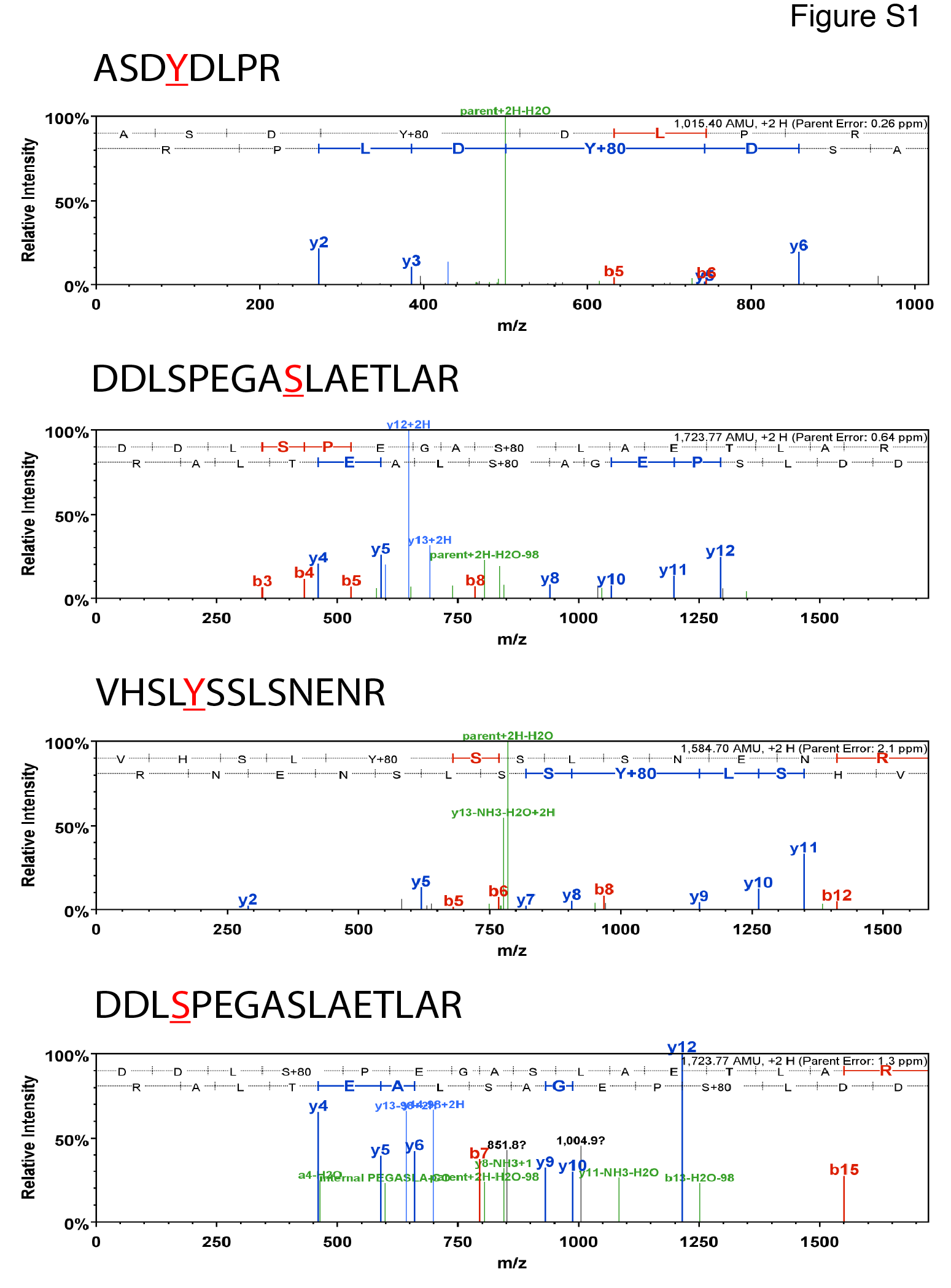

Supplement: Figure S1 — M/Z spectra of TepP phosphopeptides. Endogenous TepP was immunoprecipitated at 4 hpi. The corresponding TepP band was excised from the gel, digested with trypsin and phosphopeptides were enriched on a titanium dioxide affinity column, prior to elution and analysis by LC-MS/MS. Four phospho-peptides were detected: two phosphoserine and two phosphotyrosine. Detected peptides were shown above each spectrum and detected phosphorylation site was underlined. Y-axis is the relative intensity of peaks. X-axis is mass-to-charge ratio (m/z). (TIF) [file ppat.1003954.s001.tif]

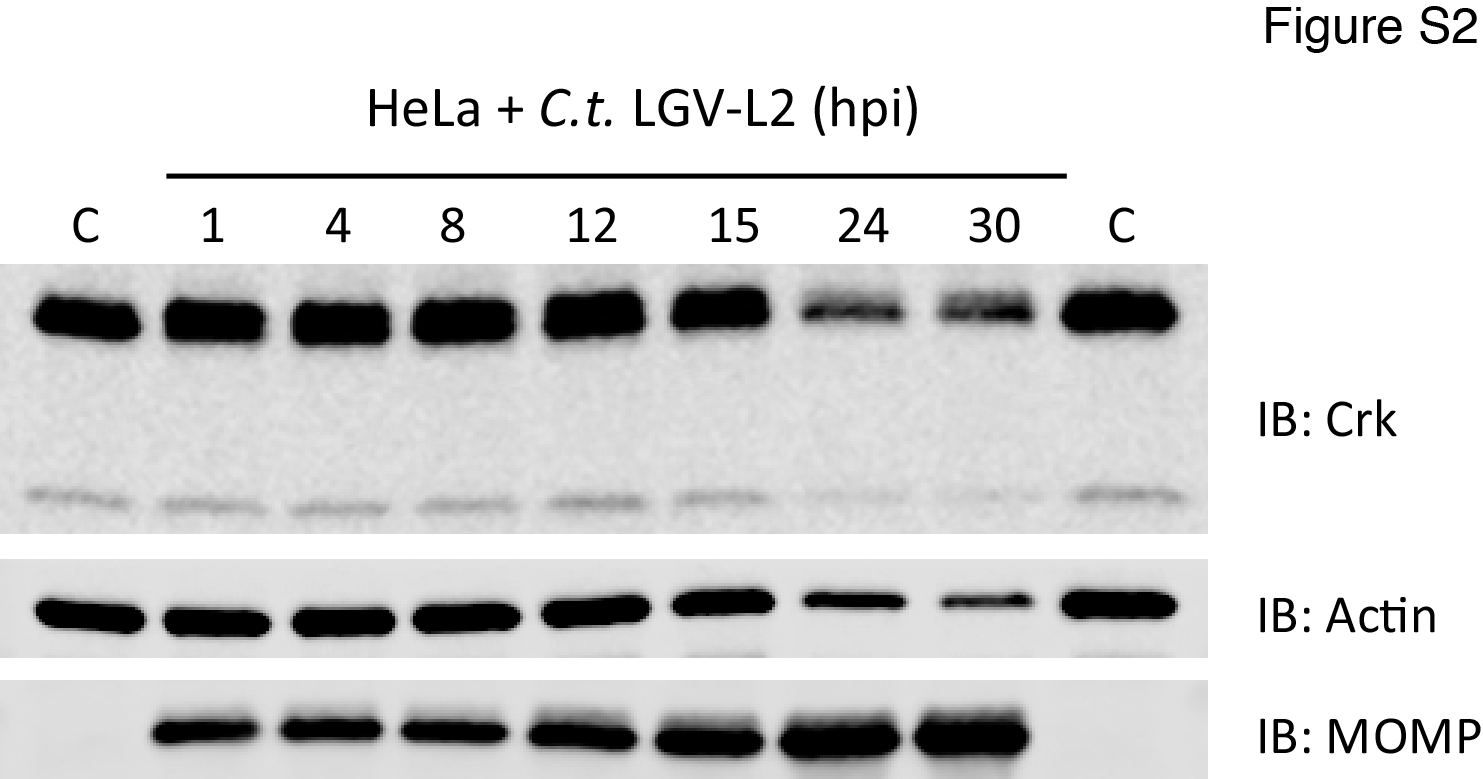

Supplement: Figure S2 — Crk levels remain constant throughout infection. Confluent monolayer of HeLa cells were infected with wild type LGV-L2 at an MOI of 50 and collected at indicated time points. Samples were subjected to immunoblot analysis with antibodies against Crk, Actin and MOMP. C: control uninfected cells. (TIF) [file ppat.1003954.s002.tif]

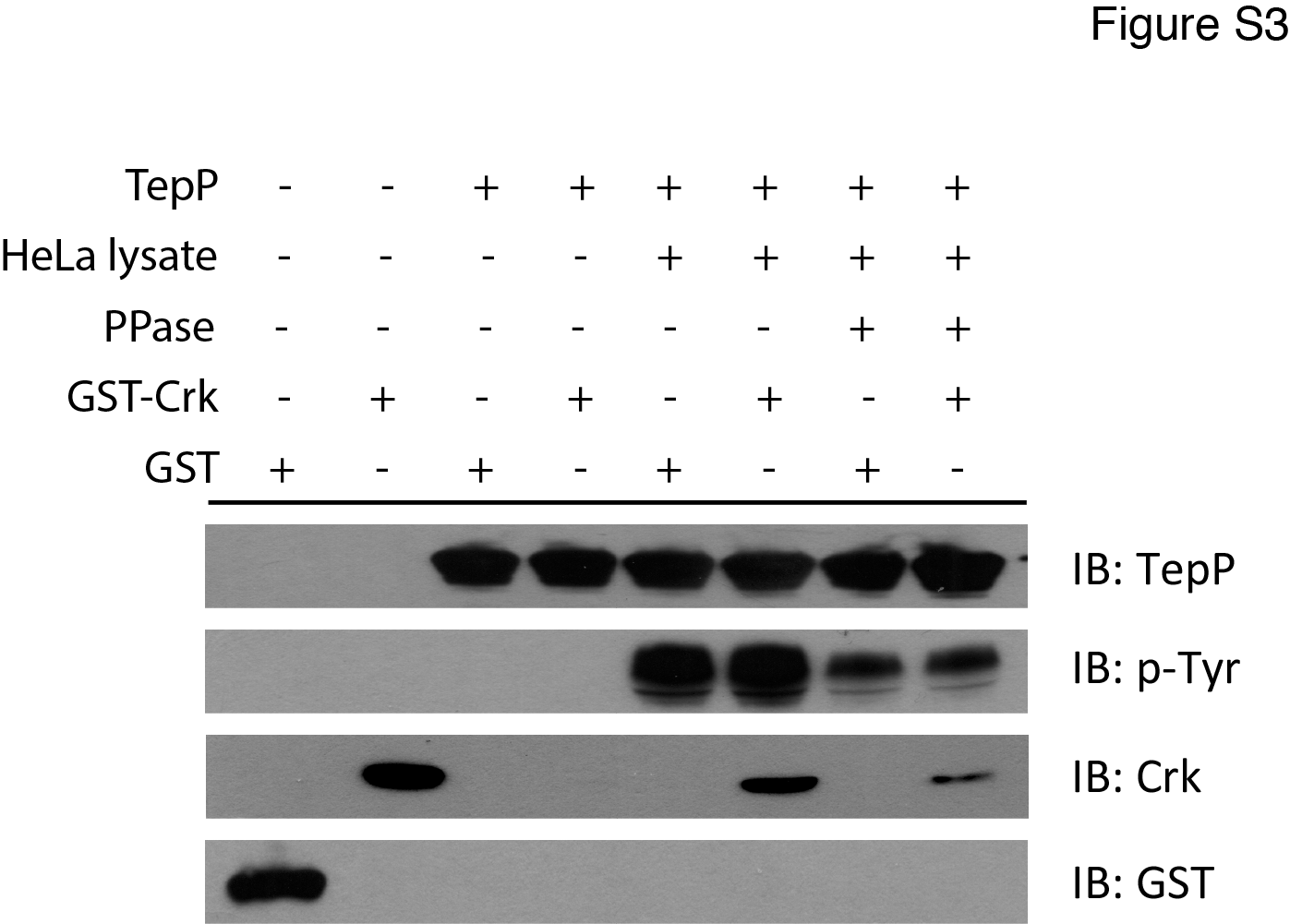

Supplement: Figure S3 — Recombinant TepP interacts with GST-Crk in a phosphorylation-dependent manner. Purified GST or GST-Crk was incubated with purified TepP-6xHis or purified TepP-6xHis that had been phosphorylated in vitro. One sample of phosphorylated TepP-6xHis was treated with Calf intestinal Alkaline Phosphatase (PPase). The efficiency of TepP co-precipitation with GST-Crk increased after in vitro phosphorylation. Dephosphorylation with PPase decreased the efficiency of GST-Crk co-precipitation. (TIF) [file ppat.1003954.s003.tif]

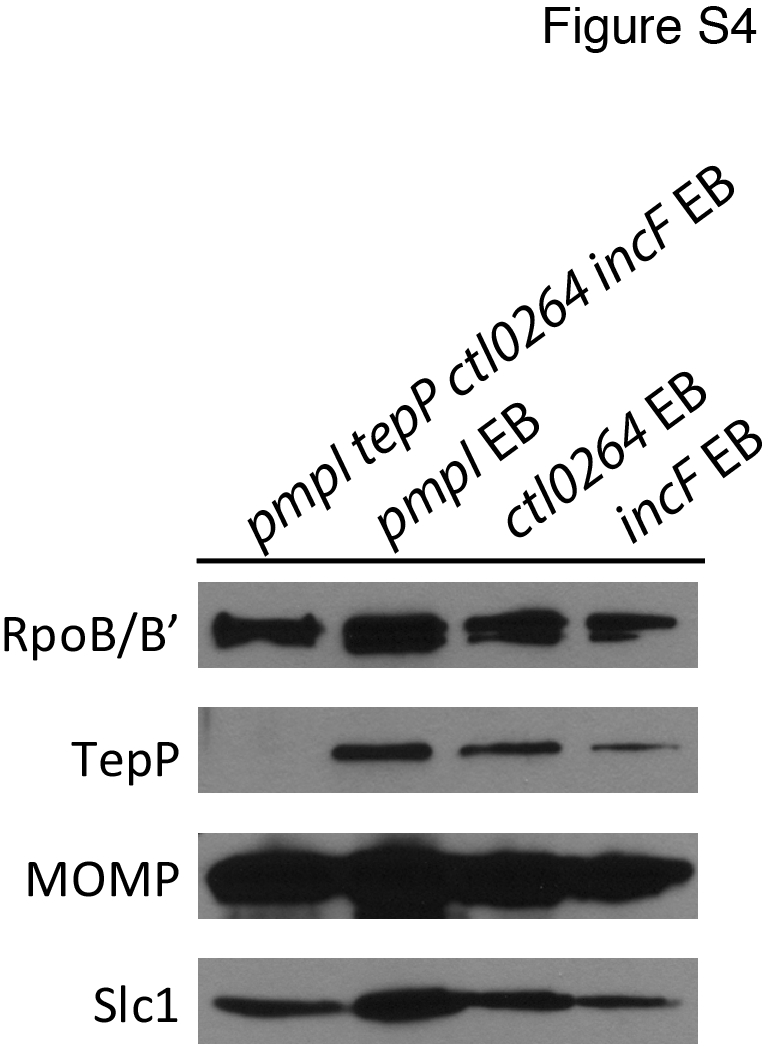

Supplement: Figure S4 — C. trachomatis recombinant harboring a tepP null allele ( tepP W103*) does not express full length TepP. EBs of four recombinants were selected from a co-infection setting between CTL2-M062 (Rifr) and a Spcr LGV-L2 variant. Recombinants were isolated by plaquing on Vero cell monolayers in the presence of rifampin and spectinomycin. Individual plaques were amplified on Vero cells, and EBs from recombinants with the genotypes shown (also see Fig. S5) were harvested and purified on density gradients. EBs were lyzed in SDS sample buffer, and subjected to immunoblot analysis with antibodies against TEPP and RpoB/B', MOMP and Slc1. (TIF) [file ppat.1003954.s004.tif]

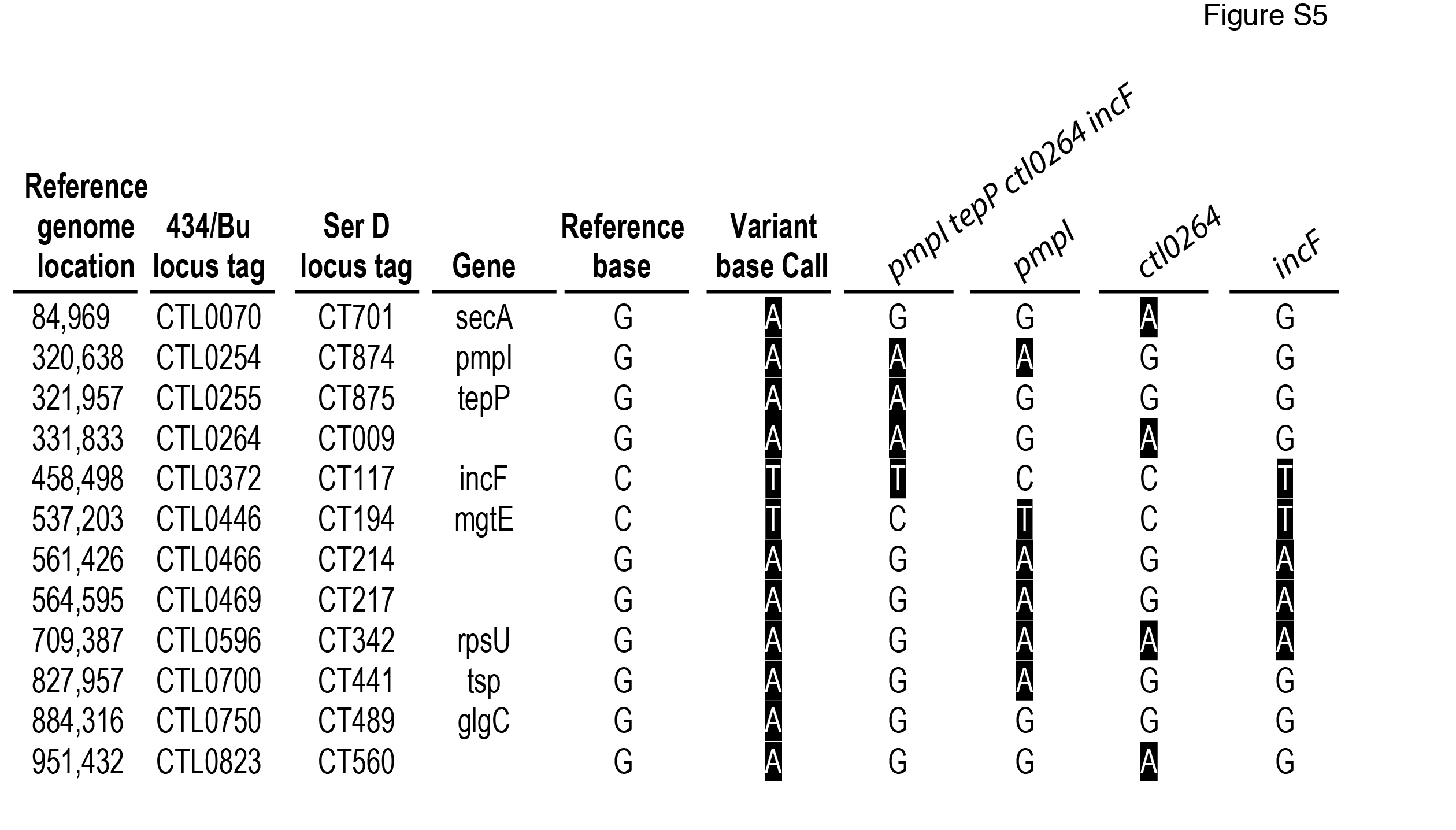

Supplement: Figure S5 — Genotype of recombinant strains harboring products of a CTL2-M062 (Rifr) and a LGV-L2 Spcr co-infection. Black shading indicates single nucleotide variants (SNV) present in CTL2-M062. Each SNV was verified by Sanger sequencing. (TIF) [file ppat.1003954.s005.tif]

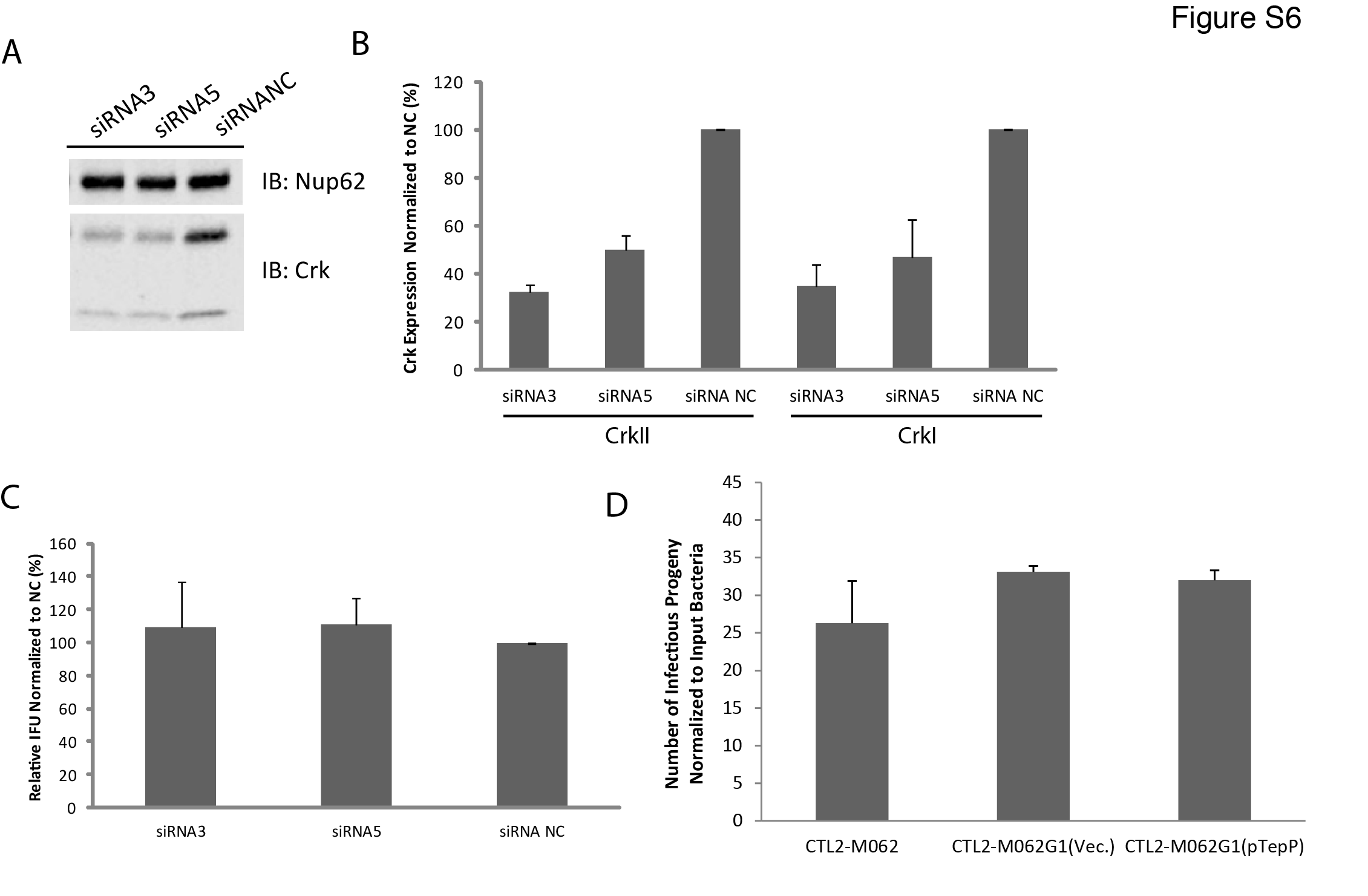

Supplement: Figure S6 — Replication potential of LGV-L2 in Crk knockdown cells and tepP mutants in epithelial cells. A) Transfection of Crk siRNAs decreased the expression level of both CrkI and CrkII in HeLa cells. Upper panel is the immunoblot analysis of HeLa cells transfected with two different Crk siRNAs (3 and 5) or negative control siRNA (NC) for 48 h. Total cell lysates were probed with anti-Crk and anti-Nup62 (loading control) antibodies. B) Quantification of siRNA-mediated decreased levels of Crk protein expression as assessed by quantitative immunoblots on a LI-COR imager. CrkI and CrkII expression level was decreased around 50% after siRNA treatment. C) Crk siRNA knockdown does not affect C. trachomatis growth as assessed by IFU assay. IFUs were normalized to growth in cells treated with control siRNA (NC). D) Comparison of IFU burst between the tepP mutant CTL2-MO62G1, and its derivatives transformed with empty vector or pTepP. HeLa cells were infected for 28 h at an MOI<1. The resulting infectious progeny were titered in HeLa cells as described in Supplemental Material and Methods, and normalized to input number of bacteria. All data shown were as means ± standard deviations from experiments performed in triplicate. (TIF) [file ppat.1003954.s006.tif]
