# Supplementary material for: The Chlamydia trachomatis Type III Secretion Chaperone Slc1 Engages Multiple Early Effectors, Including TepP, a Tyrosine-phosphorylated Protein Required for the Recruitment of CrkI-II to Nascent Inclusions and Innate Immune Signaling
Source: PLoS Pathog. 2014 Feb 20;10(2):e1003954. doi: 10.1371/journal.ppat.1003954 (PMC3930595; doi:10.1371/journal.ppat.1003954)
Supplement: Table S1 — Number of unique spectra identified by LC-MS/MS from samples immunoprecipitated with anti-Slc1 and anti-Mcsc antibodies from EBs. (DOCX) [file ppat.1003954.s007.docx]

**Supplementary Table 1:** Number of unique spectra^a^ identified by LC-MS/MS from samples immunoprecipitated with anti-Slc1 and Mcsc antibodies in Chlamydial EB*

|  |  |  |  |  | Control | | | Mcsc | | | Slc1 | | |
| --- | --- | --- | --- | --- | --- | --- | --- | --- | --- | --- | --- | --- | --- |
|  | Identified Proteins | Accession Number^b^ | M.W. | Name^c^ | Exp. 1 | 2 | 3 | Exp. 1 | 2 | 3 | Exp. 1 | 2 | 3 |
| 1 | T3S chaperone | gi\|237804393 | 18 kDa | Slc1 | 0 | 0 | 1 | 1 | 1 | 4 | 7 | 22 | 31 |
| 2 | Conserved hypothetical protein | gi\|165930024 | 35 kDa | Ct694 | 0 | 0 | 0 | 0 | 0 | 1 | 6 | 16 | 23 |
| 3 | Translocated actin-recruiting phosphoprotein | gi\|48869193 | 103 kDa | TARP | 0 | 0 | 0 | 0 | 0 | 0 | 9 | 15 | 34 |
| 4 | Conserved hypothetical protein | gi\|165930209 | 66 kDa | TEPP | 0 | 0 | 0 | 0 | 0 | 0 | 5 | 7 | 24 |
| 5 | T3S chaperone | gi\|237804605 | 19 kDa | Mcsc | 0 | 0 | 0 | 7 | 12 | 12 | 0 | 2 | 5 |
| 6 | Transaldolase | gi\|165931386 | 36 kDa | TalB | 0 | 0 | 1 | 0 | 0 | 0 | 3 | 13 | 20 |
| 7 | Conserved hypothetical protein | gi\|165930025 | 44 kDa | Ct695 | 0 | 0 | 0 | 0 | 0 | 0 | 1 | 8 | 25 |
| 8 | Putative integral membrane protein | gi\|165931439 | 61 kDa | Ct365 | 0 | 0 | 0 | 0 | 0 | 8 | 2 | 2 | 11 |
| 9 | T3S component protein | gi\|165929994 | 90 kDa | CdsD | 0 | 0 | 0 | 0 | 0 | 0 | 0 | 12 | 13 |
| 10 | Chaperone protein | gi\|237804743 | 71 kDa | DnaK | 0 | 0 | 0 | 0 | 0 | 0 | 0 | 3 | 10 |
| 11 | Serine protease | gi\|165931030 | 53 kDa | HtrA | 0 | 0 | 2 | 0 | 0 | 1 | 0 | 4 | 4 |
| 12 | Putative aminopeptidase | gi\|165931129 | 54 kDa | PepA | 0 | 0 | 0 | 0 | 0 | 0 | 0 | 5 | 3 |
| 13 | Late transcription unit B protein | gi\|237804429 | 11 kDa | Ct080 | 0 | 0 | 0 | 0 | 0 | 0 | 0 | 1 | 7 |
|  |  |  |  |  |  |  |  |  |  |  |  |  |  |
|  | |  |  |  |  |  |  |  |  |  |  |  |  |

***Results shown are from three independent experiments of IP, followed by mass spectrometry analysis using control IgG antibodies and antibodies against Mcsc or Slc1.**

***Proteins identified with less than 3 unique spectra or found to be less than 2 fold more abundant than in control IgG samples were not included. Exp -experiment**

^a^**Unique spectrum:** Two spectra are unique if they match different peptides (even if the peptides overlap), or if they match two different charge states of the same peptide, or different modified forms of the same peptide.

^b^**Accesion number for NCBI**

^c^**Gene name or CT nomenclature based on *C. trachomatis* serovar D**
