# Supplementary material for: The Chlamydia trachomatis Type III Secretion Chaperone Slc1 Engages Multiple Early Effectors, Including TepP, a Tyrosine-phosphorylated Protein Required for the Recruitment of CrkI-II to Nascent Inclusions and Innate Immune Signaling
Source: PLoS Pathog. 2014 Feb 20;10(2):e1003954. doi: 10.1371/journal.ppat.1003954 (PMC3930595; doi:10.1371/journal.ppat.1003954)
Supplement: Table S2 — Plasmid constructs used in this study. (DOCX) [file ppat.1003954.s008.docx]

**Supplementary Table 2:** Plasmid constructs used in this study.

| Vector | Insert |
| --- | --- |
|  |  |
| ***Yersinia* T3S system** |  |
| pBAD24 | Mcsc(FL) |
| pBAD24 | Slc1(FL) |
| pBAD33 | Ct694 (FL) |
| pBAD33 | Ct695(FL)-FLAG |
| pBAD33 | TepP(FL) (Ct875) |
| **GST pulldown** |  |
| pET24d | Slc1(FL) |
| pGEX | GST |
|  | GST-Ct288 (305 a.a. - 560 a.a.) |
|  | GST-TARP(FL) |
|  | GST-Ct694(FL) |
|  | GST-Ct695(FL) |
|  | GST-TepP(FL) |
| **Gel filtration** |  |
| pET24d | Slc1(FL)-6His |
|  | Slc1/TARP(FL)-6His |
|  | Slc1/Ct694(FL)-6His |
|  | Slc1/Ct695(FL)-6His |
|  | Slc1/TepP(FL)-6His |
| ***Chlamydia* Transformation** |  |
| p2TK2-SW2 | TepP(FL) |
