# Supplementary material for: The Chlamydia trachomatis Type III Secretion Chaperone Slc1 Engages Multiple Early Effectors, Including TepP, a Tyrosine-phosphorylated Protein Required for the Recruitment of CrkI-II to Nascent Inclusions and Innate Immune Signaling
Source: PLoS Pathog. 2014 Feb 20;10(2):e1003954. doi: 10.1371/journal.ppat.1003954 (PMC3930595; doi:10.1371/journal.ppat.1003954)
Supplement: Table S3 — Single nucleotide variants in strain CTL2-M062. (DOCX) [file ppat.1003954.s009.docx]

**Supplementary Table 3:** Single nucleotide variants identified in strain CTL2-M062.

| **Reference genome location** | **Reference Base** | **Variant Base Call** | **Codon**  **change** | **Amino Acid Change** | **Variant type** | **Gene** | **434/Bu locus tag** | **Ser D locus tag** | **Product** | **Coverage** | **Strand-Bias** | **Variant Frequency** |
| --- | --- | --- | --- | --- | --- | --- | --- | --- | --- | --- | --- | --- |
| 84,969 | G | A | G514A | G172R | Non synonymous | secA | CTL0070 | CT701 | preprotein translocase subunit SecA | 523 | 58% | 100% |
| 145,291 | G | A | G1797A |  | Synonymous |  | CTL0113 | CT744 | hypothetical protein | 462 | 60% | 100% |
| 192,335 | C | T | G2187A |  | Synonymous | priA | CTL0147 | CT778 | primosome assembly protein PriA | 556 | 50% | 100% |
| 192,335 | C | T | C1128T |  | Synonymous | bioF | CTL0146 | CT777 | 8-amino-7-oxononanoate synthase | 556 | 50% | 100% |
| 216,843 | C | T |  |  | Non coding |  |  |  |  | 616 | 50% | 99% |
| 320,638 | G | A | C737T | S246F | Non synonymous | pmpI | CTL0254 | CT874 | polymorphic outer membrane protein | 386 | 51% | 100% |
| 321,957 | G | A | G309A | W103* | Non sense | tepP | CTL0255 | CT875 | translocated early phospho protein | 328 | 51% | 100% |
| 331,833 | G | A | G213A | M71I | Non synonymous |  | CTL0264 | CT009 | hypothetical protein | 470 | 57% | 100% |
| 335,058 | G | A | G528A |  | Synonymous |  | CTL0267 | CT012 | putative integral membrane protein | 613 | 54% | 100% |
| 444,524 | C | T | C1650T |  | Synonymous |  | CTL0360 | CT105 | hypothetical protein | 604 | 51% | 100% |
| 458,498 | C | T | C230T | T77I | Non synonymous | incF | CTL0372 | CT117 | inclusion membrane protein F | 553 | 51% | 100% |
| 480,180 | G | A | G480A |  | Synonymous |  | CTL0395 | CT140 | exported protein | 474 | 53% | 99% |
| 514,164 | C | T | G521A |  | Synonymous |  | CTL0425 | CT140 | hypothetical protein | 659 | 50% | 100% |
| 537,203 | C | T | G541A | V181I | Non synonymous | mgtE | CTL0446 | CT194 | magnesium transport protein | 569 | 53% | 100% |
| 558,011 | C | T | C1153T |  | Synonymous | hemL | CTL0462 | CT210 | glutamate-1-semialdehyde aminotransferase | 566 | 53% | 100% |
| 561,426 | G | A | C185T | P62L | Non synonymous |  | CTL0466 | CT214 | hypothetical protein | 490 | 65% | 99% |
| 564,595 | G | A | G128A | G43D | Non synonymous |  | CTL0469 | CT217 | hypothetical protein | 486 | 52% | 100% |
| 604,651 | C | T | C471T |  | Synonymous |  | CTL0506 | CT254 | inner membrane protein | 676 | 53% | 100% |
| 689,168 | G | A | G423A |  | Synonymous | xseA | CTL0583 | CT329 | exodeoxyribonuclease VII large subunit | 572 | 58% | 100% |
| 709,387 | G | A | C140T | A47V | Non synonymous | rpsU | CTL0596 | CT342 | 30S ribosomal protein S21 | 496 | 58% | 100% |
| 726,889 | C | T | G717A |  | Synonymous |  | CTL0610 | CT356 | hypothetical protein | 393 | 55% | 100% |
| 826,893 | C | T | G243A |  | Synonymous | rpsL | CTL0698 | CT439 | 30S ribosomal protein S12 | 529 | 51% | 100% |
| 827,957 | G | A | G85A | D29N | Non synonymous | tsp | CTL0700 | CT441 | carboxy-terminal processing protease | 509 | 51% | 100% |
| 846,119 | C | T | C567T |  | Synonymous | plsC | CTL0713 | CT453 | 1-acyl-sn-glycerol-3-phosphate acyltransferase | 521 | 60% | 99% |
| 884,316 | G | A | C458T | S153L | Non synonymous | glgC | CTL0750 | CT489 | glucose-1-phosphate adenylyltransferase | 660 | 50% | 100% |
| 951,432 | G | A | G408A | M136I | Non synonymous |  | CTL0823 | CT560 | hypothetical protein | 459 | 55% | 100% |
| 123,163 | C | A | C -> A |  | Non coding |  |  |  |  | 423 | 52% | 100% |
| 127,338 | T | A | T657A |  | Synonymous |  | CTL0103 | CT734 | putative lipoprotein | 369 | 54% | 100% |
| 435,453 | G | A | C743T | A248V | Non synonymous | nusA | CTL0352 | CT097 | transcription elongation factor NusA | 630 | 55% | 100% |
| 448,523 | G | A | C110T | A37V | Non synonymous |  | CTL0364 | CT109 | hypothetical protein | 587 | 65% | 100% |
| 616,827 | C | T | G315A |  | Synonymous |  | CTL0518 | CT266 | hypothetical protein | 616 | 52% | 100% |
| 637,039 | C | T | C610T | R204C | Non synonymous | clpC | CTL0538 | CT286 | ATP-dependent Clp protease | 698 | 54% | 100% |
| 638,353 | T | C | T1924C | F642L | Non synonymous | clpC | CTL0538 | CT286 | ATP-dependent Clp protease | 548 | 56% | 100% |
| 675,519 | G^§^ | A^§^ | C1411T | H471Y | Non synonymous | rpoB | CTL0567 | CT315 | DNA-directed RNA polymerase subunit beta | 585 | 51% | 100% |
| 693,265 | G | A | G473A | S158N | Non synonymous | pykF | CTL0586 | CT332 | pyruvate kinase | 649 | 51% | 100% |
| 798,820 | G | A | G470A | G157E | Non synonymous | pmpC | CTL0671 | CT414 | polymorphic outer membrane protein | 600 | 56% | 100% |
| 929,717 | C | A | C -> A |  | Non coding |  |  |  |  | 544 | 53% | 100% |

***Single nucleotide variants in black are variants identified in CTL2M062.**

***Single nucleotide variants in gray are strain background variants differing from 434/Bu reference sequence.**

**^§^ Variant leading to rifampin resistance.**

***All non-parental non synonymous variants were verified by Sanger sequencing.**
