# Supplementary material for: The Chlamydia trachomatis Type III Secretion Chaperone Slc1 Engages Multiple Early Effectors, Including TepP, a Tyrosine-phosphorylated Protein Required for the Recruitment of CrkI-II to Nascent Inclusions and Innate Immune Signaling
Source: PLoS Pathog. 2014 Feb 20;10(2):e1003954. doi: 10.1371/journal.ppat.1003954 (PMC3930595; doi:10.1371/journal.ppat.1003954)
Supplement: Table S4 — List of genes that display a TepP-dependent regulation at 4 hpi as determined by microarray analysis. (DOCX) [file ppat.1003954.s010.docx]

**Supplementary Table 4:**  List of genes that display a TepP-dependent regulation at 4 hpi as determined by DNA Microarray analysis

|  | | | | |  |  |  |  |  |  |  |  |  |
| --- | --- | --- | --- | --- | --- | --- | --- | --- | --- | --- | --- | --- | --- |
|  |  |  |  | **p-value^b^** | **Fold-Change** |  | **p-value^b^** | **Fold-Change** |  | **p-value^b^** | **Fold-Change** |  |  |
| **Column ID^a^** | **Gene Symbol** | **Gene Title** | **RefSeq Transcript ID** | **G1V^c^ vs. G1TEPP^d^** | **G1V vs. G1TEPP** | **Description** | **Uninf^e^ vs. G1TEPP** | **Uninf vs. G1TEPP** | **Description** | **Uninf vs. G1V** | **Uninf vs. G1V** | **Description** |  |
| 204794_at | DUSP2 | dual specificity phosphatase 2 | NM_004418 | 0.001 | 1.531 | G1V up vs G1TEPP | 0.003 | 1.263 | Uninf up vs G1TEPP | 0.005 | -1.213 | Uninf down vs G1V |  |
| 222162_s_at | ADAMTS1 | ADAM metallopeptidase with thrombospondin type 1 motif, 1 | NM_006988 | 0.001 | 1.726 | G1V up vs G1TEPP | 0.021 | 1.173 | Uninf up vs G1TEPP | 0.002 | -1.472 | Uninf down vs G1V |  |
| 205027_s_at | MAP3K8 | mitogen-activated protein kinase kinase kinase 8 | NM_005204 | 0.002 | -1.857 | G1V down vs G1TEPP | 0.001 | -2.244 | Uninf down vs G1TEPP | 0.051 | -1.209 | Uninf down vs G1V |  |
| 215483_at | AKAP9 | A kinase (PRKA) anchor protein (yotiao) 9 | NM_005751 | 0.001 | -1.544 | G1V down vs G1TEPP | 0.006 | -1.301 | Uninf down vs G1TEPP | 0.019 | 1.186 | Uninf up vs G1V |  |
| 209101_at | CTGF | connective tissue growth factor | NM_001901 | 0.001 | 1.562 | G1V up vs G1TEPP | 0.015 | 1.213 | Uninf up vs G1TEPP | 0.007 | -1.288 | Uninf down vs G1V |  |
| 214329_x_at | TNFSF10 | tumor necrosis factor (ligand) superfamily, member 10 | NM_003810 | 0.003 | -1.709 | G1V down vs G1TEPP | 0.002 | -1.848 | Uninf down vs G1TEPP | 0.273 | -1.081 | Uninf down vs G1V |  |
| 207850_at | CXCL3 | chemokine (C-X-C motif) ligand 3 | NM_002090 | 0.003 | 1.570 | G1V up vs G1TEPP | 0.465 | -1.041 | Uninf down vs G1TEPP | 0.002 | -1.634 | Uninf down vs G1V |  |
| 203665_at | HMOX1 | heme oxygenase (decycling) 1 | NM_002133 | 0.003 | -1.739 | G1V down vs G1TEPP | 0.003 | -1.783 | Uninf down vs G1TEPP | 0.721 | -1.025 | Uninf down vs G1V |  |
| 202364_at | MXI1 | MAX interactor 1 | NM_005962 | 0.004 | -1.763 | G1V down vs G1TEPP | 0.003 | -1.910 | Uninf down vs G1TEPP | 0.343 | -1.083 | Uninf down vs G1V |  |
| 217502_at | IFIT2 | interferon-induced protein with tetratricopeptide repeats 2 | NM_001547 | 0.004 | -1.682 | G1V down vs G1TEPP | 0.003 | -1.768 | Uninf down vs G1TEPP | 0.496 | -1.051 | Uninf down vs G1V |  |
| 204526_s_at | TBC1D8 | TBC1 domain family, member 8 (with GRAM domain) | NM_007063 | 0.004 | -1.603 | G1V down vs G1TEPP | 0.004 | -1.576 | Uninf down vs G1TEPP | 0.776 | 1.018 | Uninf up vs G1V |  |
| 215599_at | GUSBP3 /// GUSBP9 | glucuronidase, beta pseudogene 3 /// glucuronidase, beta pseudogene 9 | NR_027386 | 0.003 | -1.636 | G1V down vs G1TEPP | 0.009 | -1.412 | Uninf down vs G1TEPP | 0.079 | 1.159 | Uninf up vs G1V |  |
| 214657_s_at | LOC100653017 /// MIR612 /// NEAT1 | uncharacterized LOC100653017 /// microRNA 612 /// nuclear paraspeckle assembly transcri | NR_002802 | 0.004 | -1.808 | G1V down vs G1TEPP | 0.008 | -1.596 | Uninf down vs G1TEPP | 0.198 | 1.133 | Uninf up vs G1V |  |
| 209102_s_at | HBP1 | HMG-box transcription factor 1 | NM_012257 | 0.008 | -1.577 | G1V down vs G1TEPP | 0.005 | -1.759 | Uninf down vs G1TEPP | 0.232 | -1.115 | Uninf down vs G1V |  |
| 212841_s_at | PPFIBP2 | PTPRF interacting protein, binding protein 2 (liprin beta 2) | NM_003621 | 0.008 | -1.756 | G1V down vs G1TEPP | 0.010 | -1.678 | Uninf down vs G1TEPP | 0.640 | 1.047 | Uninf up vs G1V |  |
| 207069_s_at | SMAD6 | SMAD family member 6 | NM_005585 | 0.007 | -1.517 | G1V down vs G1TEPP | 0.012 | -1.406 | Uninf down vs G1TEPP | 0.310 | 1.079 | Uninf up vs G1V |  |
| 219995_s_at | ZNF750 | zinc finger protein 750 | NM_024702 | 0.009 | -1.603 | G1V down vs G1TEPP | 0.019 | -1.437 | Uninf down vs G1TEPP | 0.254 | 1.115 | Uninf up vs G1V |  |
| 216015_s_at | NLRP3 | NLR family, pyrin domain containing 3 | NM_004895 | 0.008 | 1.525 | G1V up vs G1TEPP | 0.051 | 1.236 | Uninf up vs G1TEPP | 0.052 | -1.234 | Uninf down vs G1V |  |
| 204235_s_at | GULP1 | GULP, engulfment adaptor PTB domain containing 1 | NM_016315 | 0.013 | -1.505 | G1V down vs G1TEPP | 0.013 | -1.505 | Uninf down vs G1TEPP | 0.999 | 1.000 | Uninf up vs G1V |  |
| 211834_s_at | TP63 | tumor protein p63 | NM_001114978 | 0.008 | 1.514 | G1V up vs G1TEPP | 0.047 | 1.246 | Uninf up vs G1TEPP | 0.062 | -1.216 | Uninf down vs G1V |  |
| 216248_s_at | NR4A2 | nuclear receptor subfamily 4, group A, member 2 | NM_006186 | 0.034 | 1.985 | G1V up vs G1TEPP | 0.441 | -1.179 | Uninf down vs G1TEPP | 0.020 | -2.339 | Uninf down vs G1V |  |
| 204621_s_at | NR4A2 | nuclear receptor subfamily 4, group A, member 2 | NM_006186 | 0.007 | 1.965 | G1V up vs G1TEPP | 0.143 | -1.221 | Uninf down vs G1TEPP | 0.003 | -2.400 | Uninf down vs G1V |  |
| 204622_x_at | NR4A2 | nuclear receptor subfamily 4, group A, member 2 | NM_006186 | 0.041 | 1.741 | G1V up vs G1TEPP | 0.073 | -1.544 | Uninf down vs G1TEPP | 0.009 | -2.687 | Uninf down vs G1V |  |
| 214059_at | IFI44 | Interferon-induced protein 44 | NM_006417 | 0.010 | -1.847 | G1V down vs G1TEPP | 0.024 | -1.550 | Uninf down vs G1TEPP | 0.189 | 1.192 | Uninf up vs G1V |  |
| 219312_s_at | ZBTB10 | zinc finger and BTB domain containing 10 | NM_023929 | 0.017 | -1.627 | G1V down vs G1TEPP | 0.015 | -1.675 | Uninf down vs G1TEPP | 0.792 | -1.030 | Uninf down vs G1V |  |
| 204237_at | GULP1 | GULP, engulfment adaptor PTB domain containing 1 | NM_016315 | 0.014 | -1.576 | G1V down vs G1TEPP | 0.020 | -1.496 | Uninf down vs G1TEPP | 0.599 | 1.053 | Uninf up vs G1V |  |
| 206843_at | CRYBA4 | crystallin, beta A4 | NM_001886 | 0.017 | 1.562 | G1V up vs G1TEPP | 0.032 | 1.424 | Uninf up vs G1TEPP | 0.390 | -1.097 | Uninf down vs G1V |  |
| 207286_at | CEP135 | centrosomal protein 135kDa | NM_014645 | 0.020 | -1.572 | G1V down vs G1TEPP | 0.026 | -1.513 | Uninf down vs G1TEPP | 0.727 | 1.039 | Uninf up vs G1V |  |
| 209348_s_at | MAF | v-maf musculoaponeurotic fibrosarcoma oncogene homolog (avian) | NM_005360 | 0.026 | -1.619 | G1V down vs G1TEPP | 0.024 | -1.645 | Uninf down vs G1TEPP | 0.903 | -1.016 | Uninf down vs G1V |  |
| 211548_s_at | HPGD | hydroxyprostaglandin dehydrogenase 15-(NAD) | NM_000860 | 0.021 | -2.021 | G1V down vs G1TEPP | 0.032 | -1.820 | Uninf down vs G1TEPP | 0.556 | 1.110 | Uninf up vs G1V |  |
| 203914_x_at | HPGD | hydroxyprostaglandin dehydrogenase 15-(NAD) | NM_000860 | 0.049 | -1.984 | G1V down vs G1TEPP | 0.051 | -1.969 | Uninf down vs G1TEPP | 0.974 | 1.008 | Uninf up vs G1V |  |
| 204472_at | GEM | GTP binding protein overexpressed in skeletal muscle | NM_005261 | 0.042 | 1.585 | G1V up vs G1TEPP | 0.357 | -1.157 | Uninf down vs G1TEPP | 0.020 | -1.834 | Uninf down vs G1V |  |
| 205587_at | FGFR1OP | FGFR1 oncogene partner | NM_007045 | 0.019 | 1.528 | G1V up vs G1TEPP | 0.077 | 1.274 | Uninf up vs G1TEPP | 0.140 | -1.199 | Uninf down vs G1V |  |
| 203153_at | IFIT1 | interferon-induced protein with tetratricopeptide repeats 1 | NM_001548 | 0.050 | -1.510 | G1V down vs G1TEPP | 0.029 | -1.669 | Uninf down vs G1TEPP | 0.495 | -1.105 | Uninf down vs G1V |  |
| 205207_at | IL6 | interleukin 6 (interferon, beta 2) | NM_000600 | 0.050 | 2.017 | G1V up vs G1TEPP | 0.485 | -1.192 | Uninf down vs G1TEPP | 0.029 | -2.403 | Uninf down vs G1V |  |
| 204635_at | RPS6KA5 | ribosomal protein S6 kinase, 90kDa, polypeptide 5 | NM_004755 | 0.037 | -1.596 | G1V down vs G1TEPP | 0.044 | -1.547 | Uninf down vs G1TEPP | 0.827 | 1.032 | Uninf up vs G1V |  |
| 212984_at | ATF2 | activating transcription factor 2 | NM_001256090 | 0.049 | -1.560 | G1V down vs G1TEPP | 0.196 | -1.257 | Uninf down vs G1TEPP | 0.216 | 1.241 | Uninf up vs G1V |  |
|  |  |  |  |  |  |  |  |  |  |  |  |  |  |

| ^a^probeset ID on Affymetrix human genome U133A 2.0 array | |
| --- | --- |
| ^b^p-value was calculated using ANOVA analysis | |
| ^c^ CTL2-M062G1V- transformed with empty vector | |
| ^d^ CTL2-M062G1 TEPP- transformed with vector containing wild type *tepP* gene | |
| ^e^Uninf - uninfected A2EN cells |  |
