# Supplementary material for: The Chlamydia trachomatis Type III Secretion Chaperone Slc1 Engages Multiple Early Effectors, Including TepP, a Tyrosine-phosphorylated Protein Required for the Recruitment of CrkI-II to Nascent Inclusions and Innate Immune Signaling
Source: PLoS Pathog. 2014 Feb 20;10(2):e1003954. doi: 10.1371/journal.ppat.1003954 (PMC3930595; doi:10.1371/journal.ppat.1003954)
Supplement: Table S5 — Primer sequences used for Q-PCR. (DOCX) [file ppat.1003954.s011.docx]

**Supplementary Table 5.** Primer sequences used for Q-PCR

|  |  |
| --- | --- |
| Primer | Sequence (5' - 3') |
|  |  |
| IL-6 Forward | gatgagtacaaaagtcctgatcca |
| IL-6 Reverse | ctgcagccactggttctgt |
| CXCL3 Forward | aaatcatcgaaaagatactgaacaag |
| CXCL3 Reverse | ggtaagggcagggaccac |
| MAP3k8 Forward | cgcaagaggctgctgagt |
| MAP3k8 Reverse | ttcctgtgcacgaagaatca |
| IFIT1 Forward | agccagatctcagaggagcc |
| IFIT1 Reverse | ccatttgtactcatggttgctgtaa |
| IFIT2 Forward | tggtggcagaagaggaagat |
| IFIT2 Reverse | gtaggctgctctccaaggaa |
| Actin Forward | ccaaccgcgagaagatga |
| Actin Reverse | ccagaggcgtacagggatag |
| 16S rRNA Forward | ggaggctgcagtcgagaatct |
| 16S rRNA Reverse | ttacaaccctagagccttcatcaca |
|  |  |
