# Supplementary material for: The Chlamydia trachomatis Type III Secretion Chaperone Slc1 Engages Multiple Early Effectors, Including TepP, a Tyrosine-phosphorylated Protein Required for the Recruitment of CrkI-II to Nascent Inclusions and Innate Immune Signaling
Source: PLoS Pathog. 2014 Feb 20;10(2):e1003954. doi: 10.1371/journal.ppat.1003954 (PMC3930595; doi:10.1371/journal.ppat.1003954)
Supplement: Text S1 — Supporting materials and methods. (DOCX) [file ppat.1003954.s012.docx]

**SUPPLEMENTARY MATERIALS AND METHODS**

***In vitro* GST-Crk binding assay**

Hexahistidine-tagged TepP was purified on Nickel resin (Qiagen, Valencia, CA, USA) and beads with bound TepP were aliquoted into three tubes. Two tubes were incubated with HeLa cell lysates for *in vitro* phosphorylation for 1 hour at 4°C and one of the tubes was further treated with Alkaline Phosphatase, Calf Intestinal (CIP)(New England BioLabs, Ipswich, MA, USA) 50 Unit/rxn to reverse phosphorylation reactions at 37°C for 40 min in NEB buffer 3. To prepare HeLa cell lysates for *in vitro* phosphorylation, around 3x10^6^ HeLa cells were lyzed in *in vitro* kinase buffer (100 mM KCl, 10 mM HEPES, pH 7.7, 2 mM MaCl_2_ and 2 mM ATP) supplemented with PMSF, Halt phosphatase inhibitor (Pierce, Rockford, Illinois, USA) and protease inhibitor cocktail (Roche, Basel, Switzerland). Cell debris was removed by centrifugation. The last tube was incubated with *in vitro* kinase buffer only as a control. Purified GST and GST-Crk were added to tubes containing TepP, phospho-TepP or CIP-treated TepP and incubated in the cold room for 2 hours. After washing resins four times with Pierce IP lysis buffer (25 mM Tris, 150 mM NaCl, 1 mM EDTA, 1 % NP-40, 5 % glycerol; pH 7.4)(Pierce, Rockford, Illinois, USA), bound proteins were denatured in sample buffer and analyzed by immunoblots with anti-GST, -Crk, -TepP and -p-Tyr antibodies.

**Infectious Forming Unit (IFU) assay**

Confluent monolayers of HeLa cells were infected with various *Chlamydia* strains at an MOI < 1. Bacteria in infected cells were harvested at 28 hpi by hypotonic lysis as previously described [1]. Serial dilutions of the cell lysates were used to infect a new monolayer of HeLa cells and inclusions were detected by immunostaining with anti-Slc1 antibodies at 24 hpi. The number of inclusions was counted under a fluorescence microscope. The total number of IFUs obtained at 28h (output) was divided by the number of input bacteria to derive number of infectious progeny per input bacteria.

**Crk siRNA knockdowns**

HeLa cells were transfected either with siRNAs designed against Crk (Qiagen Cat. No. 1027416) or with negative control siRNA (Qiagen Cat. No. 1027280) using Lipofectamine RNAiMAX reagent (Invitrogen, Life Technologies corp., Grand Island, NY, USA). At 48 h post transfection, cells were harvested and analyzed by immunoblot with anti-Crk and anti-Nup62 (loading control) (Mab414, Covance, Princeton, NJ, USA). For IFU assay, cells were infected with wild type LGV-L2 at an MOI < 1 for 28 hours after 48 hours of siRNA transfection. IFU was determined as described above.

**Reference**

1. Nguyen BD, Valdivia RH (2013) Forward Genetic Approaches in Chlamydia trachomatis. J Vis Exp.

**SUPPLEMENTARY FIGURES**

**Figure S1**. **M/Z spectra of TepP phosphopeptides**. Endogenous TepP was immunoprecipitated at 4 hpi. The corresponding TepP band was excised from the gel, digested with trypsin and phosphopeptides were enriched on a titanium dioxide affinity column, prior to elution and analysis by LC-MS/MS. Four phospho-peptides were detected: two phosphoserine and two phosphotyrosine. Detected peptides were shown above each spectrum and detected phosphorylation site was underlined. Y-axis is the relative intensity of peaks. X-axis is mass-to-charge ratio (m/z).

**Figure S2**. **Crk levels remain constant throughout infection.** Confluent monolayer of HeLa cells were infected with wild type LGV-L2 at an MOI of 50 and collected at indicated time points. Samples were subjected to immunoblot analysis with antibodies against Crk, Actin and MOMP. C: control uninfected cells.

**Figure S3**. **Recombinant TepP interacts with GST-Crk in a phosphorylation-dependent manner.** Purified GST or GST-Crk was incubated with purified TepP-6xHis or purified TepP-6xHis that had been phosphorylated *in vitro.*  One sample of phosphorylated TepP-6xHis was treated with Calf intestinal Alkaline Phosphatase (PPase). The efficiency of TepP co-precipitation with GST-Crk increased after *in vitro* phosphorylation. Dephosphorylation with PPase decreased the efficiency of GST-Crk co-precipitation.

**Figure S4.** ***C. trachomatis* recombinant harboring a *tepP* null allele (*tepP* W103*) does not express full length TepP.** EBs of four recombinants were selected from a co-infection setting between CTL2-M062 (Rif^r^) and a Spc^r^ LGV-L2 variant. Recombinants were isolated by plaquing on Vero cell monolayers in the presence of rifampin and spectinomycin. Individual plaques were amplified on Vero cells, and EBs from recombinants with the genotypes shown (also see Fig S5) were harvested and purified on density gradients. EBs were lyzed in SDS sample buffer, and subjected to immunoblot analysis with antibodies against TEPP and RpoB/B’, MOMP and Slc1.

**Figure S5**. **Genotype of recombinant strains harboring products of a CTL2-M062 (Rif^r^) and a LGV-L2 Spc^r^ co-infection**. Black shading indicates single nucleotide variants (SNV) present in CTL2-M062. Each SNV was verified by Sanger sequencing.

**Figure S6.** **Replication potential of LGV-L2 in Crk knockdown cells and *tepP* mutants in epithelial cells**. A) Transfection of Crk siRNAs decreased the expression level of both CrkI and CrkII in HeLa cells. Upper panel is the immunoblot analysis of HeLa cells transfected with two different Crk siRNAs (3 and 5) or negative control siRNA (NC) for 48 h. Total cell lysates were probed with anti-Crk and anti-Nup62 (loading control) antibodies. B) Quantification of siRNA-mediated decreased levels of Crk protein expression as assessed by quantitative immunoblots on a LI-COR imager. CrkI and CrkII expression level was decreased around 50% after siRNA treatment. C) Crk siRNA knockdown does not affect *C. trachomatis* growth as assessed by IFU assay. IFUs were normalized to growth in cells treated with control siRNA (NC). D) Comparison of IFU burst between the *tepP* mutant CTL2-MO62G1, and its derivatives transformed with empty vector or pTepP. HeLa cells were infected for 28 h at an MOI < 1. The resulting infectious progeny were titered in HeLa cells as described in Supplemental Material and Methods, and normalized to input number of bacteria. All data shown were as means ± standard deviations from experiments performed in triplicate.

**Supplementary Table 1**. Number of unique spectra identified by LC-MS/MS from samples immunoprecipitated with anti-Slc1 and anti-Mcsc antibodies from EBs.

**Supplementary Table 2**. Plasmid constructs used in this study.

**Supplementary Table 3**. Single nucleotide variants in strain CTL2-M062.

**Supplementary Table 4.** List of genes that display a TepP-dependent regulation at 4 hpi as determined by Microarray analysis.

**Supplementary Table 5.** Primer sequences used for Q-PCR.
